# The Arabidopsis *Wall Associated Kinase-Like 10* Gene Encodes a Functional Guanylyl Cyclase and Is Co-Expressed with Pathogen Defense Related Genes

**DOI:** 10.1371/journal.pone.0008904

**Published:** 2010-01-26

**Authors:** Stuart Meier, Oziniel Ruzvidzo, Monique Morse, Lara Donaldson, Lusisizwe Kwezi, Chris Gehring

**Affiliations:** 1 Computational Bioscience Research Centre, King Abdullah University of Science and Technology, Thuwal, Kingdom of Saudi Arabia; 2 Department of Biotechnology, University of the Western Cape, Bellville, South Africa; University of Melbourne, Australia

## Abstract

**Background:**

Second messengers have a key role in linking environmental stimuli to physiological responses. One such messenger, guanosine 3′,5′-cyclic monophosphate (cGMP), has long been known to be an essential signaling molecule in many different physiological processes in higher plants, including biotic stress responses. To date, however, the guanylyl cyclase (GC) enzymes that catalyze the formation of cGMP from GTP have largely remained elusive in higher plants.

**Principal Findings:**

We have identified an Arabidopsis receptor type wall associated kinase–like molecule (AtWAKL10) as a candidate GC and provide experimental evidence to show that the intracellular domain of AtWAKL10_431–700_ can generate cGMP *in vitro*. Further, we also demonstrate that the molecule has kinase activity indicating that AtWAKL10 is a twin-domain catalytic protein. A co-expression and stimulus-specific expression analysis revealed that *AtWAKL10* is consistently co-expressed with well characterized pathogen defense related genes and along with these genes is induced early and sharply in response to a range of pathogens and their elicitors.

**Conclusions:**

We demonstrate that AtWAKL10 is a twin-domain, kinase-GC signaling molecule that may function in biotic stress responses that are critically dependent on the second messenger cGMP.

## Introduction

The intracellular second messenger guanosine 3′,5′-cyclic monophosphate (cGMP) has been shown to be an important signaling molecule that controls a broad range of physiological responses in eukaryotes and prokaryotes [1]. Cyclic GMP is generated following stimulus-induced activation of guanylyl cyclase (GC) enzymes that catalyze the synthesis of cGMP from guanosine 5′-triphosphate (GTP) [1]. In animals, two main classes of GCs are known to exist, a plasma membrane localized receptor (rGC) class that is activated by ligands and a soluble cytoplasmic (sGC) form that is predominately activated by nitric oxide (NO) [2].

In higher plants, cGMP has been shown to be an essential signaling molecule in many diverse physiological processes [3] including NO-dependent signaling [4], biotic [5] and abiotic stress responses [6], transcriptional regulation [7], as well as gravitropic [8] and plant hormone-dependent responses [9]. Furthermore, significant and transient increases in intracellular cGMP levels have also been reported in response to plant natriuretic peptides (PNPs) [10], NaCl and drought stress [11], ozone [6] and pathogen challenge [12].

In tobacco, cGMP has been implicated as an essential downstream signaling molecule in NO-mediated pathogen defense responses and is required for the induced expression of the defense-related genes, *phenylalanine ammonia lyase* (*PAL*) and *pathogenesis-related (PR)-1*, and also for activation of PAL enzyme activity [5] which generates precursors for phenylpropanoid and thus salicylic acid (SA) biosynthesis [13]. Consistent with this, NO treatment of tobacco leaves induced endogenous SA accumulation [5] and it has been proposed that increased cGMP levels induce increases in cytosolic calcium levels which in turn activate SA biosynthesis and accumulation resulting in activation of SA signaling pathways [14]. Additionally, in *Arabidopsis thaliana* cell cultures, cGMP has been shown to be required for NO-induced cell death in response to infection with the avirulent bacterial pathogen, *Pseudomonas syringae* pv. *maculicola avrRpm1* [15]. While these studies used GC inhibitors and a cell-permeable cGMP analogue (8-Bromo-cGMP) to help elucidate the role of cGMP in defense responses, only recently have endogenous cGMP levels been shown to increase in response to pathogens. Both virulent and avirulent strains of *P. syringae* were shown to induce an increase in cGMP generation with the *avrB* strain inducing a more rapid response [12].

**Figure 1:**
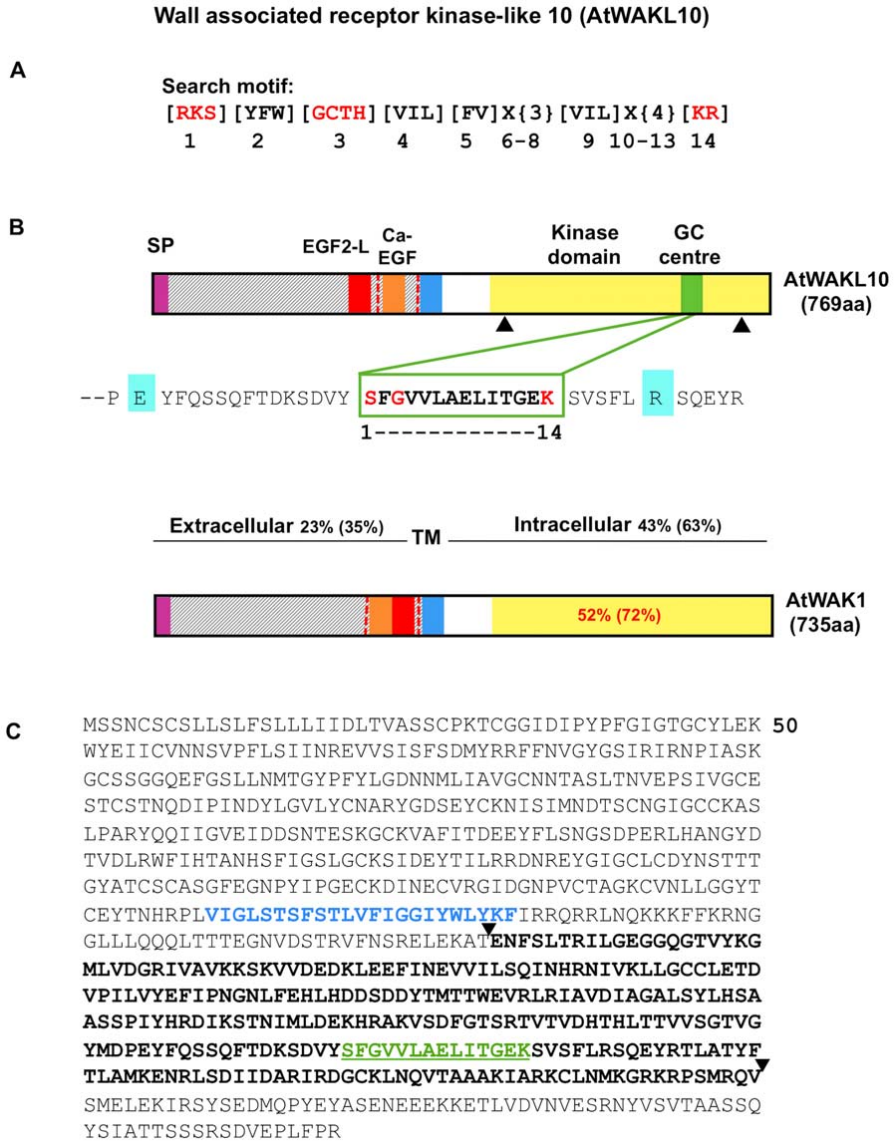
Structural features of the AtWAKL10 protein. (**A**) The 14 aa long search motif generated based on conserved and functionally-assigned aa in the catalytic centres of annotated GCs. Amino acid substitutions in the search motif are in square brackets ([ ]); X represents any aa and curly brackets ({ }) define the number of aa. Amino acids in red are functionally assigned residues, [1] hydrogen bonds with the guanine; [3] confers substrate specificity for GTP; [7] binds to the dimer interphase and [14] stabilizes the transition state from GTP to cGMP. (**B**) Domain organization of AtWAKL10 and AtWAK1 illustrating the location of the predicted signal peptides (SP), extracellular EGF-like domains, TM domains and kinase domains and for AtWAKL10 the GC centre imbedded in the kinase domain. The percentages indicate the determined aa identity and (similarity) between AtWAKL10 and AtWAK1 at the indicated regions. The dashed red vertical lines represent intron locations. The two black triangles demarcate the truncated cytosolic fragment of the AtWAKL10_431–700_ protein that was expressed as a recombinant. The Arg (R, highlighted in aquamarine) C-terminal of the catalytic centre is a putative metal binding residue and the N-terminal Glu (E, highlighted in aquamarine) is the putative pyrophosphate binding residue. (**C**) Predicted aa sequence of AtWAKL10. The aa in blue represent the TM domain that separates the extracellular domain from the cytosolic domain. Sequences in bold and demarcated by the two triangles represent the sequence of the recombinant AtWAKL10_431–700_ protein that was expressed for functional testing. The GC domain is marked in green letters and underlined.

Despite the increasing body of functional evidence that indicates cGMP is an essential signaling molecule in many physiological processes in plants [3], to date little is known about cGMP-generating GCs in higher plants. The identification of GCs in higher plants is complicated by the fact that BLAST searches with annotated GCs from either higher or lower eukaryotes fail to identify any matches. This suggests that higher plants have evolved unique GC molecules where only the catalytic centre [16] may show any degree of conservation [17]. In light of this, a search motif for GCs was designed based on the functionally assigned amino acids (aa) in the catalytic centre of annotated GCs (Figure 1A) in lower and higher eukaryotes [17]. This motif identified seven candidate GCs in Arabidopsis and the first of these, AtGC1 (At5g05930), was tested and confirmed to have GC activity *in vitro*. One of the remaining GC candidates is the wall associated kinase (WAK)-like 10 protein (AtWAKL10, At1g79680).

WAK and WAKLs have been identified in many plant species [18] and the Arabidopsis genome has been reported to encode 26 WAK and WAK-like (WAKL) proteins [18]–[20]. AtWAKL10, along with all other annotated WAKL genes, was identified in reiterative database searches (BLAST) using WAK1 cDNA or WAK1 proteins as queries and most WAKLs, including AtWAKL10, were found to have similar intron-exon and functional domain organization to WAK1 [18].

The *WAK/WAKL* genes are typically predicted to encode a class of receptor-like protein kinases that posses a transmembrane (TM) domain, a cytoplasmic serine/threonine kinase (STK) domain and an extracellular region that is tightly associated with the cell wall and contains several epidermal growth factor (EGF) repeats that may act as ligand binding domains [20]. The AtWAKL10 protein has previously been predicted to contain an extracellular calcium-binding EGF-like domain and a degenerate EGF2-like domain [18]. The extracellular domains of WAK/WAKL proteins have been reported to be less well conserved than their cytoplasmic domains [21]–[23] and it was proposed that these variations in combination with their specific expression patterns may provide Arabidopsis with the ability to detect and respond to a diverse array of ligands [18].

Studies have indicated that some WAKs may be involved in pathogen defense responses. In Arabidopsis the expression of *WAK1* was shown to be induced, in a non-expressor of PR genes (NPR)-1 dependent manner, by the *P. syringae* pathogen as well as by exogenous SA application and this induced expression is required to protect plants from high and potentially lethal levels of SA [24]. In addition, *WAK1* transcription is up-regulated by systemic acquired resistance (SAR) inducing conditions [25] and by the fungal pathogen *Alternaria brassicicola* and defense related signaling molecules including methyl jasmonate (MeJA) and ethylene (Eth) [26]. Furthermore, WAKs are required for cell expansion during elongation [27] and the extracellular domain of the WAK1 protein has been shown to bind extracellular localized molecules including a glycine-rich secreted protein (AtGRP-3) [28], pectin, polygalacturonic acid (PGA, a commercial source of homogalacturonan) and oligogalacturonides (OGs) that are in a specific calcium-induced conformation [23]. To date however, little is known about the specific functional role of AtWAKL10 or the biological processes in which AtWAKL10 functions.

Here we demonstrate that the intracellular domain of the purified recombinant AtWAKL10 protein can catalyze the formation of cGMP *in vitro* suggesting that it is a functional GC. Additionally, we show that this domain also has *in vitro* kinase activity indicating that it is a twin-domain signaling molecule. A comprehensive co-expression and stimulus-specific transcriptional analysis provides evidence to indicate that AtWAKL10 has an important functional role in early biotic stress responses in Arabidopsis. The implications of these findings and the possible biological roles of this receptor kinase are discussed.

## Results

### AtWAKL10 Sequence Comparison with AtWAK1

The predicted aa sequence of AtWAKL10 was compared to that of AtWAK1 since AtWAK1 is the most characterized WAK protein in Arabidopsis. The analysis indicated that the predicted AtWAKL10 protein has a similar mass and similar structural features to AtWAK1 (Figure 1B) as has been previously reported [18].

The extracellular region of AtWAKL10 contains the consensus sequence pattern of a EGF2-like (EGF2-L) domain (aa 305 to 321; (CxCx(2)[GP][FYW]x(4,8) C); Prosite: PS01186) and a calcium-binding EGF-like (Ca-EGFL) domain (aa 323 to 351; ([DEQN]x[DEQN](2)Cx(3,14)Cx(3,7)Cx[DN]x(4)[FY]xC); Prosite: PS01187) that are in the reverse order of AtWAK1 as has typically been reported for other AtWAKL proteins (Figure 1B) [18]. Also similar to other AtWAKL proteins, the EGF2-L domain was found to be slightly degenerate in that it contains an extra aa between the final Cys residues (x(9) instead of ×(4,8)). The functional consequences of the degenerate EGF domains are unclear since they have not been well characterized in plants.

The degree of region similarity determined is typical of comparisons between other AtWAK and AtWAKL proteins in that the intracellular regions are more conserved than the extracellular regions (Figure 1B) [18], [21], [22].

It is relevant to note that the corresponding extracellular region of AtWAKL10 contains short basic aa sequences that are similar to those determined to mediate homogalacturonan binding in AtWAK_167–254_ [29]. As with AtWAK1, this region in AtWAKL10 also contains a number of conserved cysteine residues (10 compared to 11 in AtWAK1) that may be relevant in AtWAK1 homogalacturonan interactions [29]. A basic cysteine-rich protein has previously been shown to bind pectin through ionic interactions [30] and the cysteine-rich domains were proposed to form a carbohydrate-reception pocket similar to that found in some plant lectins [31]. In AtWAK1, it was suggested that the conserved cysteines of the homogalacturonan-binding domain are involved in the formation of a 3D structure that exposes the basic arginine and lysine residues allowing them to interact in a calcium-induced conformation [29].

### *In Vitro* Enzyme Activity of AtWAKL10 Recombinant Protein

#### Recombinant protein purification

**Figure 2:**
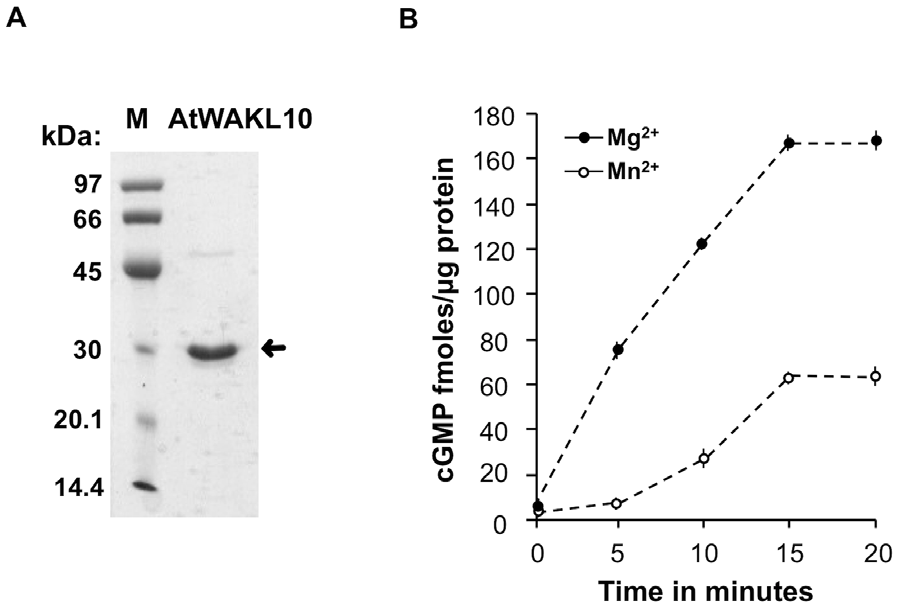
Expression of purified recombinant AtWAKL10 and determination of GC activity. (**A**) SDS-PAGE of the purified recombinant AtWAKL10_431–700_ protein, where M represents the low molecular weight marker while the arrow is marking the recombinant protein band. (**B**) Cyclic GMP generated following incubation of 10 µg of the purified recombinant AtWAKL10_431–700_ protein in a reaction system containing 50 mM Tris-HCl; pH 8.0, 1 mM GTP, 2 mM IBMX and either 5 mM Mg^2+^ or 5 mM Mn^2+^. The reaction was performed for the indicated time points at room temperature and cGMP levels were determined by enzyme immunoassay. The error bars represent the standard error of the mean (SEM) (n = 3).

The AtWAKL10 protein was previously identified as a candidate GC molecule using a 14 aa long search motif that was designed based on conserved sequences in the catalytic centers of GCs from lower and higher eukaryotes [17] (Figure 1A). In order to test if the intracellular domain of the AtWAKL10 protein can function as a GC, a truncated 270 aa recombinant protein, AtWAKL10_431–700_, was made that harbors the kinase and the imbedded predicted GC domain (Figure 1C). The purified AtWAKL10_431–700_ recombinant protein was determined to have a molecular mass of approximately 30 kDa (Figure 2A) that corresponds well with the predicted mass of 30.4 kDa for the truncated protein.

#### GC activity of AtWAKL10 protein

The purified AtWAKL10_431–700_ protein was tested for its ability to generate cGMP *in vitro*. The GC assays performed with purified AtWAKL10_431–700_ showed a time dependent increase in cGMP generation with a preference for Mg^2+^ over Mn^2+^ as a cofactor for its activity (Figure 2B). In the presence of Mg^2+^, the cGMP concentration increased from almost non-detectable levels at time 0 min to peak at around 150 fmoles cGMP/µg protein at 15 min and remained elevated at this level at 20 min (Figure 2B).

**Figure 3:**
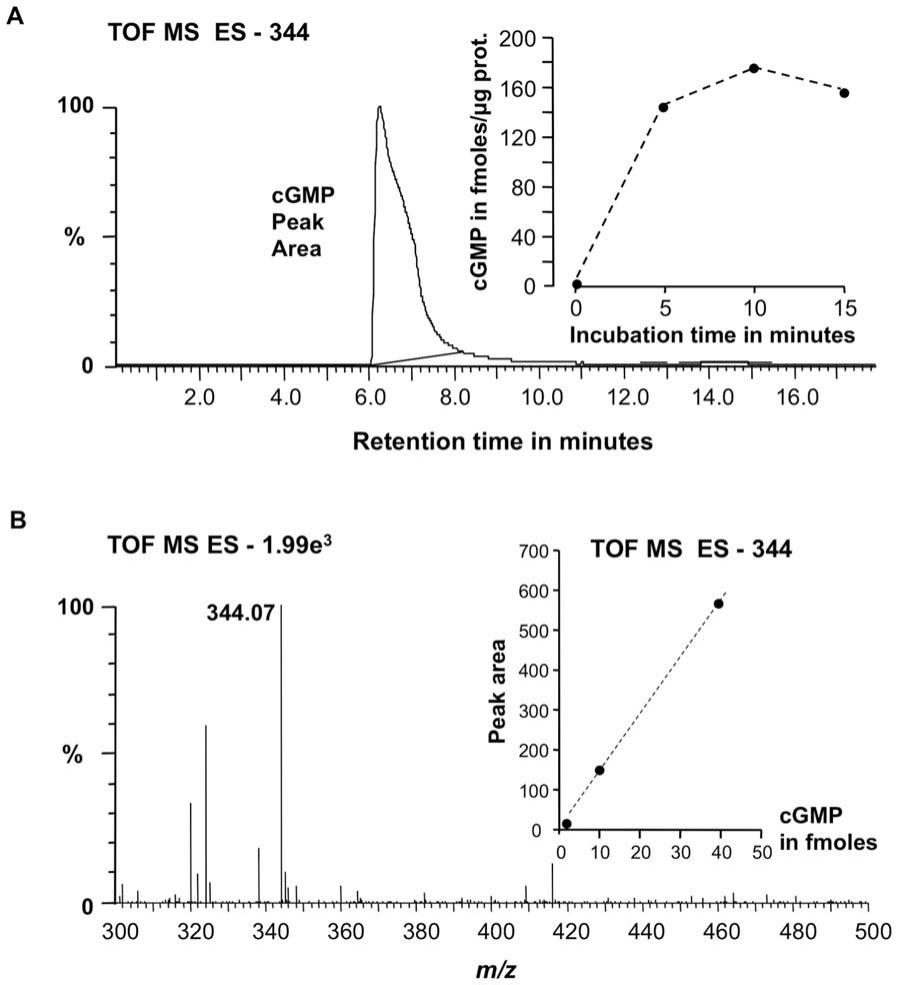
Confirmation of cGMP levels using mass spectrometry. (**A**) Extracted mass chromatogram of the *m/z* 344 [M-1]^−1^ ion of cGMP generated by recombinant AtWAKL10_431–700_ after 15 min. Inset: Incubation time course. (**B**) Mass of the resultant peak in the chromatogram. The inset represents the calibration curve with 1.25, 10 and 40 fmoles of cGMP. The experiment was performed three times and the figure is representative of a typical response.

Cyclic GMP levels were also measured by mass spectroscopic analyses to verify the result obtained by enzyme immunoassay (Figure 3). The system was calibrated by extracting mass chromatograms at *m/z* 344 [M-1]^−1^ for increasing concentrations of cGMP (Figure 3B inset). In Figure 3A, the mass peak area was generated following incubation of 10 µg of recombinant protein with the substrate GTP for 15 min in the presence of 5 mM Mg^2+^. The inset shows that recombinant protein in the presence of Mg^2+^ generates increasing amounts of cGMP in a time dependent manner with a maximum of >150 fmoles/µg protein reached after 10 min. The cGMP mass peak is resolved (Figure 3B) and we have ascertained that the reaction mix in the absence of the recombinant protein or the recombinant protein without the reaction mix did not yield mass peak signals (result not shown).

**Figure 4:**
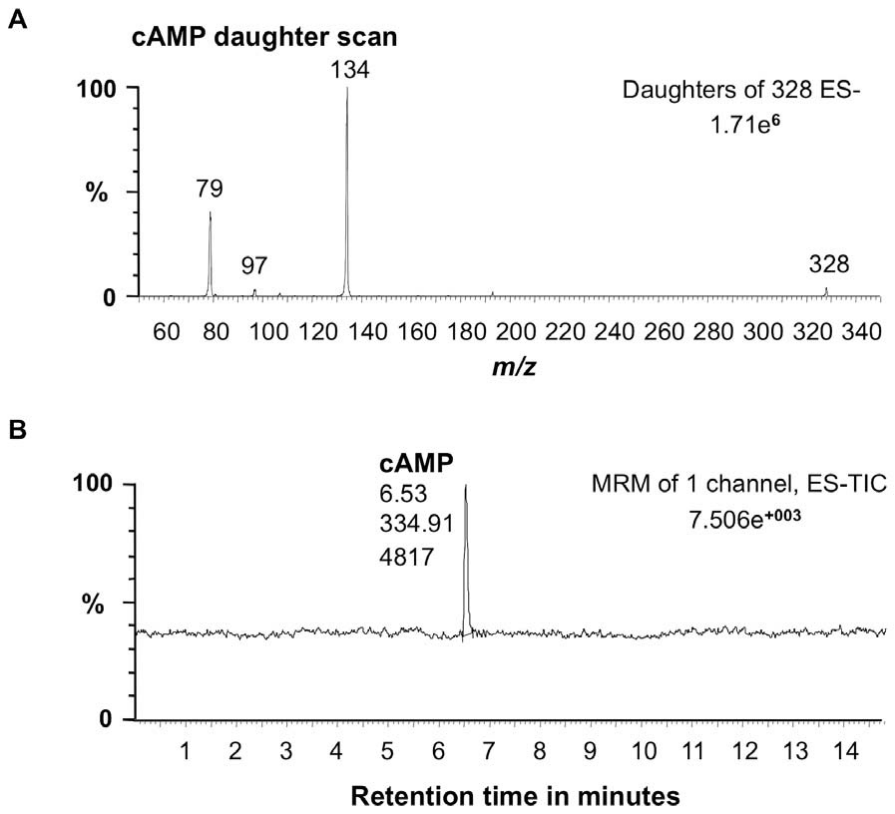
Testing the recombinant AtWAKL10 for adenylyl cyclase activity. (**A**) Extracted mass chromatograms of the *m/z* 328 [M-1]^−1^ ion of cAMP and daughter ions generated by 10 µg of recombinant AtWAKL10_431–700_ with ATP instead of GTP as the substrate, after 15 min at room temperature (24°C). (**B**) Quantified peak areas of the resultant cAMP chromatograms. The experiment was performed four times and the figure is representative of a typical response.

In order to test the predicted substrate preference, cAMP generation following incubation of 10 µg of the recombinant protein with the substrate ATP was also measured using this method (Figure 4). Firstly, cAMP (molecular mass: 329.206) and daughter ions were obtained (Figure 4A), and secondly we quantified cAMP peak areas generated by recombinant protein after 15 min in the presence of 5 mM Mg^2+^ (Fig 4B). The result indicated that <10 fmoles cAMP/µg protein was generated thus confirming specificity for GTP as the substrate.

#### Kinase activity of AtWAKL10 protein

**Figure 5:**
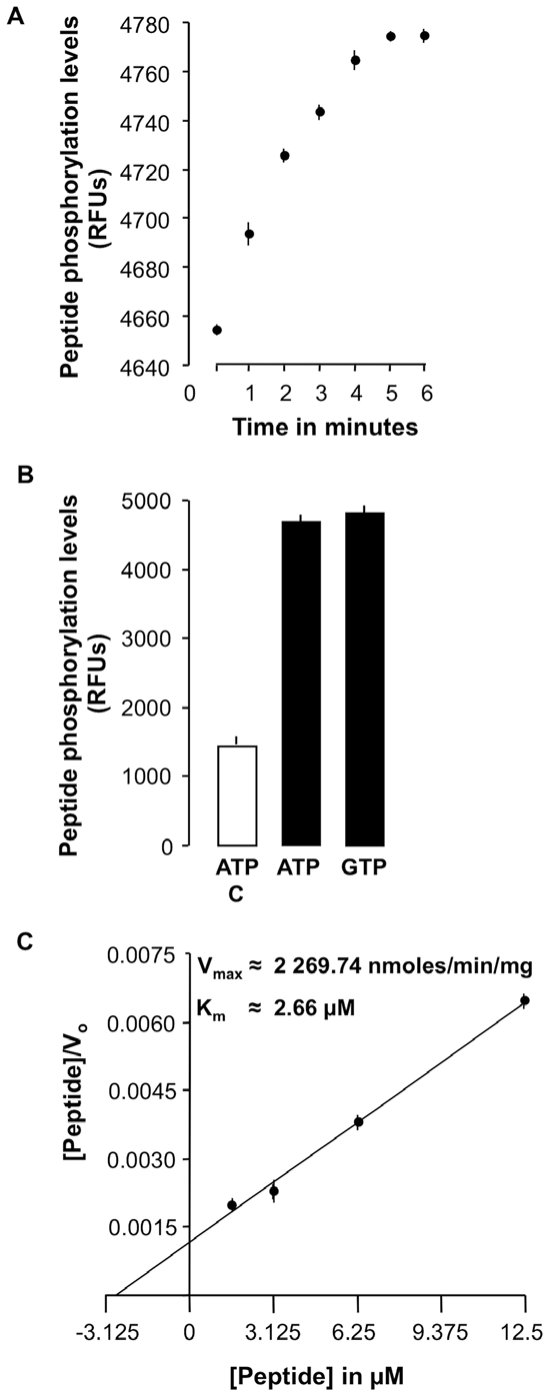
Determination of the kinase activity of the recombinant AtWAKL10. (**A**) The calibration curve obtained was produced in the presence 0.1 ng recombinant AtWAKL10_431–700_ in a reaction system containing 1 mM ATP, 0.2 mM DTT, and 25 µM Ser/Thr-peptide. Phosphorylation was quantified using the Omnia™Ser/Thr-Recombinant system at the indicated time points at 30°C (n = 3). (**B**) Kinase activity of AtWAKL10_431–700_ with either ATP or GTP as the substrate. The unfilled bar is a control which contained no recombinant protein (n = 3). (**C**) Hanes plot for the determination of the reaction kinetics of the recombinant AtWAKL10_431–700_ indicates the initial velocities for a number of Ser/Thr-peptide concentrations (1.563, 3.125, 6.25, and 12.5 µM) (n = 3). Estimated values for the kinetic constants (K_m_ and V_max_) for the recombinant AtWAKL10 protein were derived from this plot. K_m_ was determined as the negative value of the x-intercept (x = −K_m_, when y = 0) of the linear fit of the data, while V_max_ was calculated from the y-intercept (y = K_m_/V_max_, when x = 0). Error bars represent the SEM.

The kinase activity of AtWAKL10_431–700_ was also assessed using an *in vitro* Omnia™ Ser/Thr-Recombinant system (BioSource) (Figure 5). Firstly, an optimum calibration curve for the kinase activity was obtained using 0.1 ng recombinant AtWAKL10 (Figure 5A). The kinetic parameters determined for AtWAKL10_431–700_ were a V_max_ of 2269.74 nmoles/min/mg protein and a K_m_ value of 2.66 µM (Figure 5C). These phosphorylation kinetics are in the same range as those previously determined for the recombinant mouse cAMP-dependent protein kinase (V_max_ = 3700 nmoles/min/mg protein, Km = 1.8 µM) using the same method [32], [33] suggesting a relatively comparable level of Ser/Thr kinase activities in plants and animals. The results therefore confirm that in addition to harboring a functional GC domain, the recombinant AtWAKL10_431–700_ also has a functional kinase domain thus making it a twin-domain molecule.

### Computation Based Functional Analysis of AtWAKL10

A computational based co-expression and stimuli specific expression analysis was performed, following a previously described protocol [34], [35], in an attempt to elucidate the biological processes in which AtWAKL10 may function. In eukaryotes, it is widely accepted that cellular responses require the coordinated participation of multiple gene products [36] and genes that are confirmed to be co-expressed often have functional relationships, including physical interactions between their proteins [37]–[39]. Thus, insights into cellular processes in which an unknown gene may function can be gained if it is consistently co-expressed with genes that have well defined roles in specific functional processes.

#### AtWAKL10 co-expression analysis

An expression correlation analysis was performed across a large number (322) of diverse microarray experiments to determine the level that *AtWAKL10* is co-expressed with other genes represented on the ATH1-22K full genome microarray chip. This analysis revealed that *AtWAKL10* is highly co-expressed, with a number of genes in the Arabidopsis genome with the top 50 genes having a Pearson correlation coefficient (*r*-value) ranging between 0.910 and 0.810 (Table 1). In total, 516 genes (2.2%) had an *r*-value >0.6 while only 259 (1.1%) had an *r*-value>0.7 which supports the specificity of the analysis since it indicates that *AtWAKL10* is co-expressed with only a select group of genes. The top 50 most co-expressed genes (hereby referred to as the *AtWAKL10-*Expression Correlated Gene Group (ECGG)-50) were selected for further analysis since their correlation values were high and this number was considered a good representative sample size for functional enrichment and promoter analysis. A detailed functional description of the co-expressed genes is presented in Text S1 (Supporting Information) and an extended list of the top 100 correlated genes and additional genes of interest are presented in Table S1 (Supporting Information).

**Table 1:** Top 50 genes that are expression correlated with *AtWAKL10* (AT1G79680).

Rank	Locus and GO annotation	*r*	Description
1	AT5G65600	0.913	Legume lectin family protein kinase (PK) family protein
2	AT4G21390	0.907	S-locus lectin PK family protein
3	AT3G21230	0.895	4-coumarate-CoA ligase 5
4	AT4G11170^DR^	0.894	Disease Resistance Protein (TIR-NBS-LRR class)
5	AT5G67340	0.893	Armadillo/beta-catenin repeat family protein
6	AT1G26380	0.877	FAD-binding domain-containing protein
7	AT1G57650^ DR^	0.869	Disease Resistance Protein (NBS-LRR class)
8	AT2G35980^DR,RBS,ROO^	0.868	NHL10 (NDR1/HIN1-LIKE-10)
9	AT3G09410	0.867	Pectin acetylesterase family protein
10	AT1G15520^RBS,PM^	0.866	Pleiotropic Drug Resistance (PDR)-12, ABC transporter.
11	AT1G61560^DR,RBS,ROO,PM^	0.865	Mildew Resistance Locus O -6 (MLO6)
12	AT3G11840	0.862	U-box domain-containing protein
13	AT1G19180	0.862	Jasmonate-Zim-Domain protein 1 (JAZ1).
14	AT1G76070	0.861	Similar to syringolide-induced protein 14-1-1
15	AT3G26830^RBS,ROO^	0.859	Phytoalexin deficient 3 (PAD3), camalex-biosynth.
16	AT3G43250	0.858	Cell cycle control protein
17	AT1G51890	0.858	Leucine-rich repeat PK, Serine/threonine PK
18	AT4G25030	0.857	Expressed protein
19	AT3G63380	0.856	Calcium-transporting ATPase, PM-type
20	AT1G28190	0.855	Expressed protein
21	AT3G53600	0.854	Zinc finger (C2H2 type) family protein
22	AT5G67080	0.852	Similar to mitogen-activated PKKK 20 (MAPKKK20)
23	AT1G77500	0.850	N-terminal protein myristoylation
24	AT5G48400	0.848	Glutamate receptor family protein (GLR1.2)
25	AT4G17500	0.845	Ethylene-Response-Factor -1A (*ERF-1A*)
26	AT5G64890	0.844	Elicitor peptide 2 precursor (PROPEP2)
27	AT1G66090^DR^	0.843	Disease Resistance Protein (TIR-NBS class)
28	AT1G71100	0.842	Ribose 5-phosphate isomerase
29	AT1G74360	0.841	LRR-TM PK (BRI1-LIKE 2)
30	AT4G39030^DR,RBS,ROO^	0.841	SID-1/ EDS5
31	AT1G29690	0.841	CAD1, neg. reg. SA-med. pathway
32	AT4G11370	0.837	Zinc finger (C3HC4-type RING finger) family protein
33	AT1G26420	0.834	FAD-binding domain-containing protein
34	AT2G18690	0.834	Expressed protein
35	AT3G25780^DR,RBS,ROO,PM^	0.830	Allene Oxide Cyclase 3 (AOC3), JA-biosynth.
36	AT1G22400	0.829	UDP-glucoronosyl/UDP-glucosyltransferase
37	AT2G15390	0.828	Xyloglucanfucosyltransferase, putative (FUT4)
38	AT3G52400^DR,RBS,ROO,PM^	0.828	Syntaxin of Plants(SYP)-122 (SYP122) PM
39	AT1G57630^DR^	0.825	Disease Resistance Protein (TIR class)
40	AT5G26920	0.825	Calmodulin-Binding Protein 60g (CBP60g)
41	AT5G38710	0.824	Prolineoxidase, osmotic stress-responsive e
42	AT5G12340	0.823	Expressed protein;
43	AT3G45060	0.821	High-affinity nitrate transporter 2.6 (NRT2.6)
44	AT4G18170	0.820	WRKY28 transcription factor
45	AT5G05730^RBS, ROO^	0.819	Anthranilate Synth. Alpha subunit -1 (ASA1), tryp-biosyn.
46	AT2G32140^DR^	0.819	Disease Resistance Protein (TIR class)
47	AT5G44990	0.819	Similar to intracellular chloride channel
48	AT4G34390	0.816	Extra-Large GTP 2 binding protein (XLG2)
49	AT3G09010	0.815	PK family protein
50	AT4G33430^DR,RBS,ROO,PM^	0.810	BAK1

Abbreviations for indicated GO terms:

DR = defence response (GO:0006952); RBS = response to biotic stimulus; (GO:0009607); ROO = response to other organism (GO:0051707); PM = plasma membrane (GO:0005886).

#### Functional enrichment analysis of the *AtWAKL10*-ECGG50

The high expression correlation of *AtWAKL10* with genes in the ECGG50 indicates that AtWAKL10 may function in similar biological processes to these genes. The *AtWAKL10*-ECGG50 was therefore subjected to a functional enrichment analysis using “Fatigoplus” [40] to determine if there was any enrichment in functional terms associated with the group and thus link AtWAKL10 to these functions. The Gene Ontology (GO) analysis revealed that AtWAKL10 is annotated to be part of the endomembrane system, have kinase activity and function in protein amino acid phosphorylation. This analysis further revealed that the *AtWAKL10*-ECGG50 contained a significant enrichment in genes annotated to function in a number of biological processes related to pathogen defense, including at Level 3, Defense Response (DR, 11 genes, adjusted p-value = 2.5×10^−5^) and Response to Biotic Stimulus (RBS, 9 genes, adjusted p-value = 1.6×10^−4^) while at Level 4 there is an enrichment in the term Response to Other Organisms (ROO, 8 genes, adjusted p-value = 1.5×10^−3^) (Table 1, Table S2 (Supporting Information)). In the cellular component category at Level 5, there is enrichment in genes associated with the Plasma Membrane (PM, 5 genes; adjusted p-value = 2.6×10^−2^) and all of these genes are also annotated to function in RBS.

The PM plays a key role in defense by acting as a physical barrier to microbe penetration and additionally, along with the cell wall, is the first site of interaction and detection of foreign molecules [41]. Accordingly, the co-expressed PM biotic stress related genes include those that function in microbe interactions and defense signaling including, *BRASSINOSTEROID INSENSITIVE ASSOCIATED RECEPTOR KINASE* (*BAK*)-1 (*r* = 0.810, At4g33430) [42], [43], the *ELICITOR PEPTIDE PRECURSOR 2* (*PEP2*, *r* = 0.844, At5g64890) and *3 (PEP3*, *r* = 0.708, At5g64905) paralogs and their receptor *PEPR-1* (*r* = 0.777, At1g73080) [44], *CHITIN ELICITOR RECEPTOR KINASE (CERK)-1* (*r* = 0.749, At3g21630) [45], [46] and *MILDEW RESISTANCE LOCUS* (*MLO*)-6 (*r* = 0.865, At1g61560) [47].

Other PM related defense genes include those involved in pre-invasive apoplastic defense responses which attempt to inhibit pathogen penetration by poisoning the apoplast and strengthening the cell wall through the secretion of antimicrobial [48] and cell wall related proteins and compounds [49] respectively. These include *PLEIOTROPIC DRUG RESISTANCE* (*PDR*)-12 (*r* = 0.866, At1g15520) [50] and essential genes of the SNARE machinery such as *SYNTAXIN OF PLANTS* (*SYP*)-122 (*r* = 0.828, At3g52400), *PENETRATION* (*PEN*)-1 syntaxin (*SYP121*, *r* = 0.695, At3g11820), *SYNAPTOSOMAL-ASSOCIATED PROTEIN* (*SNAP*)-33 (*r* = 0.722, At5g61210) and *VESICLE-ASSOCIATED MEMBRANE PROTEIN* (*VAMP*)-722 (*r* = 0.734, At2g33120) [49].

Other genes potentially involved in microbe recognition and defense signaling include the two most highly correlated genes which are lectin protein kinases (At5g65600, *r* = 0.914 and At4g21390, *r* = 0.907) [51], [52] and five annotated Disease Resistance Proteins (*R*-proteins) that typically, indirectly or directly, detect specific pathogen secreted effectors and activate effector-triggered immune (ETI) responses [53].

It is of particular significance that *AtWAKL10* is co-expressed with genes involved in the biosynthesis and regulation of the major pathogen defense signaling molecules SA and jasmonic acid (JA). These include for SA, *SA INDUCTION DEFICIENT (SID)-1* (*r* = 0.841, At4g39030) [54], *SID-2/ISOCHORISMATE SYNTHASE (ICS)-1* (*r* = 0.810, At1g74710) [55], *CALMODULIN-BINDING PROTEIN* (*CBP)-60g* (*r* = 0.825, At5g26920) [56], and *CONSTITUTIVELY ACTIVE CELL DEATH (CAD)-1* (*r* = 0.841, At1g29690) [57]. Co-expressed genes involved in the biosynthesis of JA include *ALLENE OXIDE CYCLASE (AOC)-3* (*r* = 0.830, At3g25780), *OXOPHYTODIENOATE REDUCTASE (OPR)-3* (*r* = 0.747, At2g06050) and *LIPOXYGENASE (LOX)-3* (*r* = 0.704, At1g17420) [58].

*AtWAKL10* is also consistently co-expressed with genes encoding enzymes that function in the biosynthesis of camalexin, which is the main antimicrobial phytoalexin in Arabidopsis and is synthesized in response to a broad range of biotrophic and necrotrophic pathogens [59]. These include the tryptophan biosynthesis genes, *ANTHRANILATE SYNTHASE ALPHA (ASA)-1* (*r* = 0.819, At5g05730), *TRYPTOPHAN SYNTHASE ALPHA (TSA)-1* (*r* = 0.731, At3g54640), and *INDOLE-3-GLYCEROL PHOSPHATE SYNTHASE* (*IGPS*) (*r* = 0.799, At2g04400) and *PHYTOALEXIN DEFICIENT (PAD)-3* (*r* = 0.859, At3g26830) [59].

A number of correlated genes have also been implicated to function in regulating the transcription of defense related genes including two WRKY family transcription factors (TFs) (*WRKY28*, *r* = 0.820, At4g18170 and *WRKY15*, *r* = 0.790, At2g23320) [60]. The ETHYLENE RESPONSE FACTOR (ERF)-1A TF (*r* = 0.845, At4g17500) activates transcription of genes that contain GCC boxes (AGCCGCC) in their promoters which have been associated with defense transcriptome including several *PR* genes [61]. The *JASMONATE-ZIM-DOMAIN PROTEIN (JAZ)-1* (*r* = 0.862, At1g19180) gene encodes a negative regulator of the MYC2 TF [62] which differentially regulates two classes of JA regulated genes and is a negative regulator of defense genes that function in resistance to *Botrytis cinerea* including those involved in tryptophan metabolism [63].

Finally, a number of calcium transporting and sensing molecules are also highly correlated with *AtWAKL10* which is relevant since rapid increases in cytosolic calcium concentrations have been shown to occur and to be required for activation of downstream defense signaling following pathogen recognition [64].

#### Stimulus-specific expression profile of *AtWAKL10*-ECGG50

**Figure 6:**
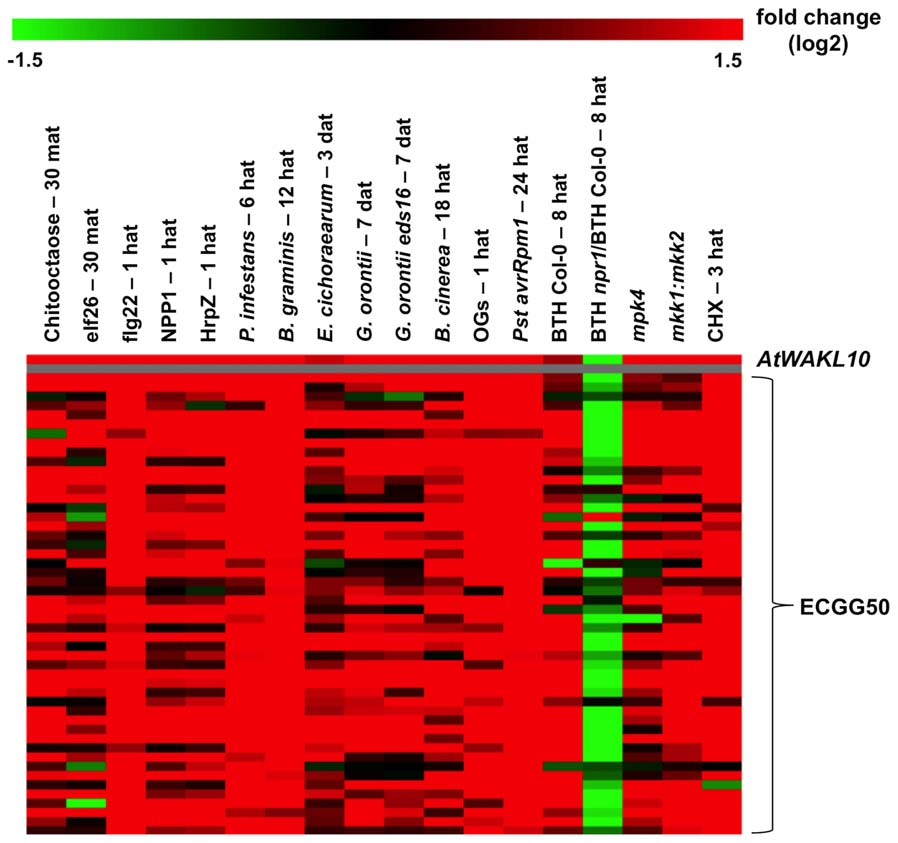
Heatmap illustrating fold change in expression of *AtWAKL10-*ECGG50 in response to select conditions. A heatmap was constructed to illustrate the fold change (log2) in expression of *AtWAKL10* and all genes in the ECGG in response to selected microarray experiments. The experiments presented include; chitooctaose (30 minutes after treatment (mat), GSE8319), elf26 (30 mat, E-MEXP-547), flg22 (1 hour after treatment (hat), NASC-409), NPP1 (1 hat, GSE5615), HrpZ (1 hat, GSE5615), *P. infestans* (6 hat, NASC-123), *B. graminis h* (12 hat, GSE12856), *E. cichoracearum* (3 days after treatment (dat), GSE431), *G. orontii* (7 dat, Col-0 and *eds16/ics1* mutant, GSE13739), *B. cinerea* (18 hat, NASC-167), OG (1 hat, NASC-409), *Pst avrRpm1* (24 hat, NASC-120), BTH treatment (BTH vs. untreated (Col-0) and BTH (*npr1)* vs. BTH (Col-0), 8 hat, NASC-392), *mpk4* (At4g01370, E-MEXP-174), *mkk1* (At4g26070) and *mkk2* (At4g29810) double mutant (GSE10646), and CHX (3 hat, NASC-189). Details of the microarray experimental conditions are presented in Text S2 (Supporting Information).

**Figure 7:**
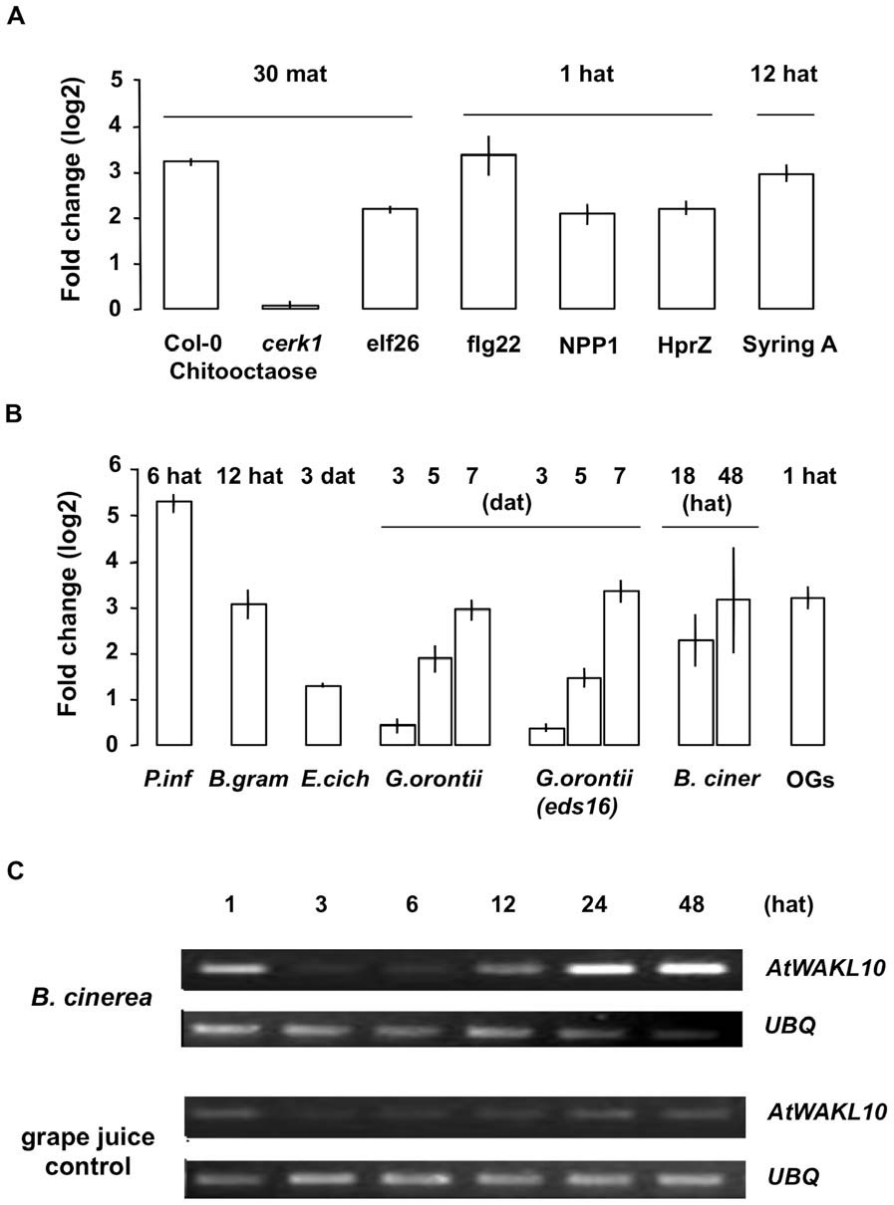
Expression of *AtWAKL10* following pathogen and elicitor challenge. (**A**) Fold change in *AtWAKL10* expression following incubation with the pathogen elicitors, chitooctaose (Col-0 and *cerk1* (At3g21630) mutant, 30 mat, GSE8319); elf26 (30 mat, E-MEXP-547), flg22 (1 hat, NASC-409), NPP1 (1 hat, GSE5615), HrpZ (1 hat, GSE5615) and Syringolin A (12 hat, E-MEXP-739) as determined from microarray experiments. (**B**) Fold change in *AtWAKL10* expression following challenge with *P. infestans* (6 hat, NASC-123), *B. graminis h* (12 hat, GSE12856), *E. cichoracearum* (3 dat, GSE431), *G. orontii* time course (Col-0 and *eds16/ics1* mutant, 3, 5 and 7 dat, GSE13739), *B. cinerea* (18 and 48 hat, NASC-167) and OG treatment (1 hat, NASC-409) as determined from microarray experiments. (**C**) Semi-quantitative RT-PCR gel image illustrating *AtWAKL10* expression over time following inoculation with 5×10^4^ spores/mL of *B. cinerea* relative to the grape juice control treatment. Expression of *AtWAKL10* was induced by *B. cinerea* biphasically at 1 and notably at 24 and 48 hat. *UBQ* was used as the “housekeeping” gene to ensure that there was equal amount of template in each sample.

**Figure 8:**
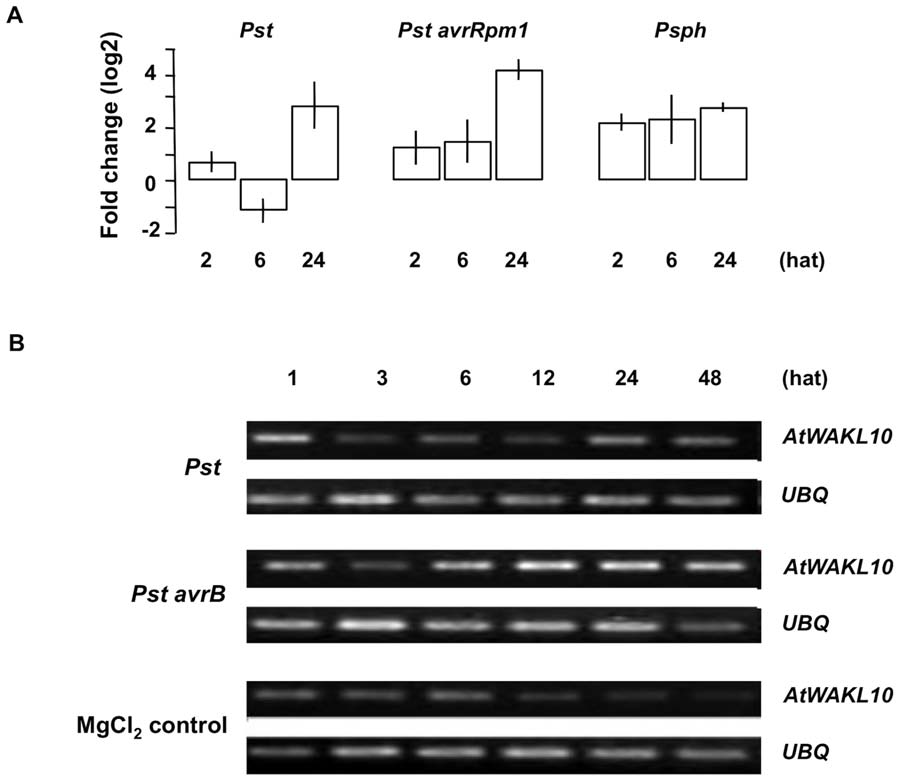
Expression of *AtWAKL10* following challenge with different strains of the bacterial *P. syringae* pathogen. (**A**) Fold change in *AtWAKL10* expression following challenge with various strains of the bacterial *P syringae* pathogen including virulent (*Pst*), avirulent (*Pst avrRpm1*), and non-host pv. *phaseolicola* (*Psph*) as determined from microarray experiments (NASC-120). (**B**) Semi-quantitative RT-PCR gel image illustrating *AtWAKL10* expression over time following inoculation with virulent (*Pst)* and the avirulent (*Pst avrB)* strains of *P. syringae* relative to the control inoculation with 10 mM MgCl_2_. Expression of *AtWAKL10* was induced by *Pst* and *Pst avrB* infection. In both cases a biphasic pattern of expression is apparent with the induced expression being faster and stronger in response to the avirulent pathogen as is expected for the compatible interaction. *UBQ* was used as the “housekeeping” gene to ensure that there was equal amount of template in each sample.

**Figure 9:**
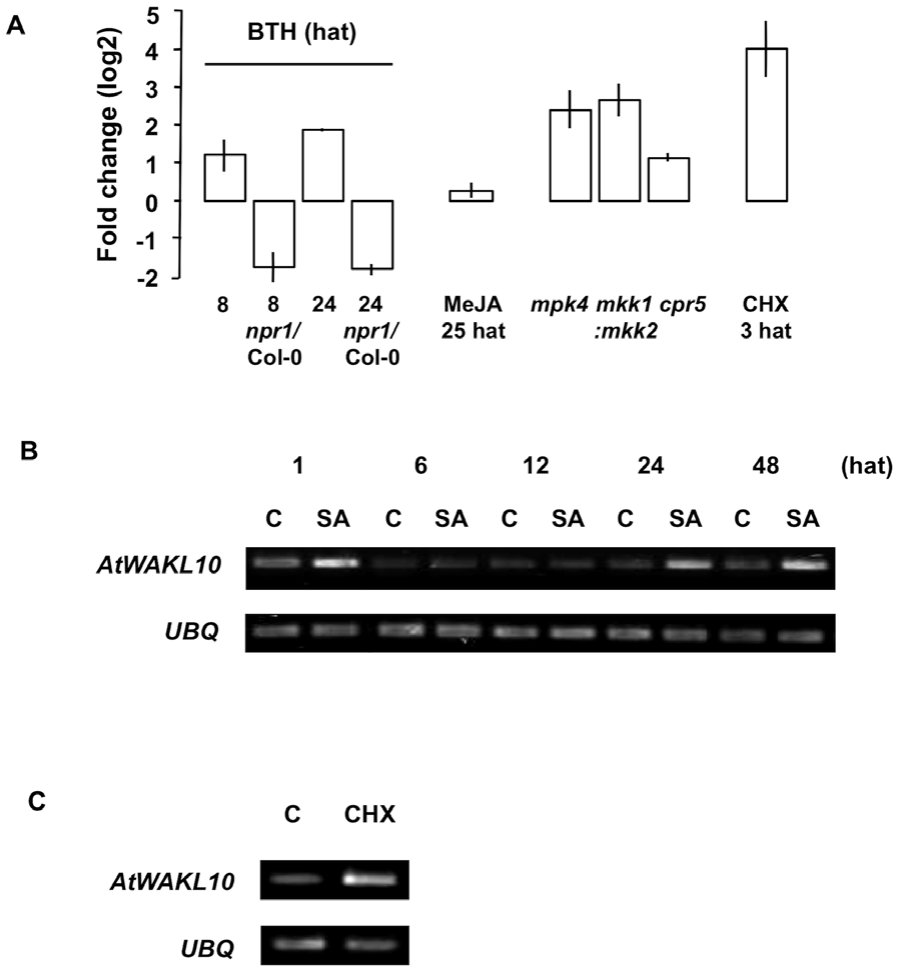
Fold change in expression of *AtWAKL10* after chemical treatment and in pathogen related mutants. (**A**) Fold change in *AtWAKL10* expression following treatment with the functional synthetic SA analogue, BTH treatment of Col-0 and *npr1* genotypes (8 and 24 hat, NASC-392), MeJA (25 hat, NASC-415) and CHX (3 hat, NASC-189) as determined from microarray experiments. Fold change in *AtWAKL10* expression was also determined from microarray experiments in a number of pathogen-related mutants including *mpk4* (E-MEXP-174), *mkk1mkk2* double mutant (GSE10646) and *cpr5* (At5g64930, GSE5745). (**B**) Semi-quantitative RT-PCR confirmed the induction of *AtWAKL10* expression in response to 10 µM CHX (3 hat) relative to the DMSO control (C). *UBQ* was used as the “housekeeping” gene to ensure that there was equal amount of template in each sample.

An *in-silico* global expression analysis was subsequently performed for the *AtWAKL10-*ECGG50 to identify specific experimental conditions that induce differential expression of the genes. In accordance with the co-expression and GO analysis, the heat maps generated from the microarray expression analysis revealed that the transcription of *AtWAKL10* and the *AtWAKL10*-ECGG50 are generally collectively induced in response to a range of pathogens, pathogen elicitors, pathogen related mutants and pathogen signaling molecules (Figure 6). To improve resolution, the expression of *AtWAKL10* alone in response to additional treatments and time course studies is presented in Figures 6–9. Unless otherwise stated, all stated fold changes in the text are log2 values.

The expression of *AtWAKL10* and the *AtWAKL10*-ECGG50 was induced early and sharply in response to well characterized microbe-associated molecular patterns (MAMPs) (Figure 6 and 7A) including, chitin, which is a major component of fungal cell walls and insect exoskeletons (chitin oligosaccharides; +3.47, 30 minutes after treatment (mat)), in a CERK1*-*dependent manner, the fully active N-terminal bacterial EF-Tu derivative, elf26 (+2.34, 30 mat) [65], and the bacterial flagellin derived peptide, flg22 (+3.60, 60 mat) [66]. The expression of the gene set was also induced by other pathogen derived elicitors including, bacterial secreted harpin, hypersensitive reaction and pathogenicity (Hrp)-Z (+2.37) [67] and syringolin A (+3.18) [68] and the oomycete-derived necrosis-inducing *Phytophthora* protein 1 (NPP1) (+2.23) [69].

Consistent with the induction of the gene group in response to a broad range of pathogen derived elicitors, the expression of *AtWAKL10* and the ECGG50 was also induced following inoculation with bacterial, fungal and oomocyte pathogens including both biotrophs and nectrotrophs (Figures 6–8). Interestingly, the induction of *AtWAKL10*, and the ECGG50 in general, in response to the non-host fungus *B. graminus* f. sp. *hordei.* (*h*) (+2.90, 12 hours after treatment (hat)) and the non-host oomycete *P. infestans* (+5.03, 6 hat) occurred earlier and was stronger than that induced by the virulent powdery mildew fungi *G. orontii* (+1.78, 5 days after treatment (dat)) and *E. cichoracearum* (+1.23, 3 dat) (Figures 6 and 7B).

This was also observed in the microarray time course following inoculation with the bacterial pathogen *P. syringae*, with the non-host pv. *phaseolicola* ((*Psph*)+2.34, 2 hat) inducing an earlier and sustained increase at all time points relative to untreated controls, compared to the virulent pv. *tomato* DC3000 (*Pst*) strain (Figure 8A). The avirulent *Pst avrRpm1* strain also induced an increase 2 hat (+1.30) that was sustained 6 hat before being sharply increased 24 hat (+4.48). The earlier induction in *AtWAKL10* expression in response to avirulent compared to the virulent strain was confirmed using semi-quantitative RT-PCR (Figure 8B).

In line with the pathogen dependent induction of *AtWAKL10* and the ECGG50, microarray studies showed that the expression of the genes was induced by the functional synthetic SA analogue, benzothiadiazole S-methylester (BTH) (+1.59, 8 and 24 hat) in an NPR1-dependent manner (Figure 9A). The induction of *AtWAKL10* by SA was experimentally confirmed with semi-quantitative RT-PCR (Figure 9B). The induction in response to BTH was not as high as that observed following pathogen infection suggesting that there are additional factors required for the full activation of these genes in response to pathogens. The expression of the *AtWAKL10*-ECGG50 was also induced in a number of pathogen related mutants that have elevated levels of SA including, *mitogen activated protein kinase (mpk)-4* (+2.04) [70], *constitutive expression of pathogenesis related genes* (*cpr*)*-5* (+0.97) [71] and the *mitogen-activated protein kinase kinase (mkk)-1* and *mkk2* double mutant (*mkk1:mkk2*) (+2.25) [72]. In contrast, however, the *G. orontii* time course revealed that the induction of the gene set was largely unchanged when performed in the SA-deficient *eds16/ics1* mutant [46] (Figure 7B) indicating that the *G. orontii* induced expression of the *AtWAKL10*-ECGG50 occurs independently of SA. The expression of the gene group was generally unaltered following MeJA treatment (Figure 9A).

Inoculation with the necrotrophic fungus, *B.cinerea,* induced a strong up-regulation of the *AtWAKL10*-ECGG50 at both 18 (+2.16) and 24 (+2.99) hat (Figure 7B). The induction for *AtWAKL10* was confirmed with semi-quantitative RT-PCR which additionally showed an increase in expression at 12 hat (Figure 7C). The expression of *AtWAKL10* was also strongly induced following challenge with OGs (+3.03, 1 hat) which are endogenous elicitors of defense responses that are released from the plant cell wall after the partial degradation of pectin by hydrolytic polygalaturonase (PGs) enzymes that are secreted from pathogens including *B. cinerea* [73]. The OGs have previously been shown to induce a variety of plant host defenses and enhance resistance to *B. cinerea* in a PAD3-dependent manner and induce a similar set of genes as those induced following *B. cinerea* infection [74].

Finally, the protein synthesis inhibitor, cycloheximide (CHX) also induced a marked and largely universal induction of the *AtWAKL10-*ECGG50 (Figure 6 and 9A). This complements the early induction observed in response to pathogens and their elicitors and indicates that their transcription is negatively regulated by rapidly turned-over repressor proteins or by transcript degrading enzymes [75]. The ability to induce expression of the gene group independent of *de novo* protein synthesis concurs with the definition of immediate early response genes [76] that are believed to play important roles in the early regulation of defense responses [77].

#### Promoter analysis of *AtWAKL10*-ECGG50

The highly correlated expression of the *AtWAKL10*-ECGG50 indicates that these genes are under common regulatory control and thus may share common *cis-*regulatory elements in their promoter regions. The promoter regions of these genes were therefore analyzed for the presence of enriched *cis*-elements using the Athena [78] and POBO [79] promoter analysis programs. The Athena analysis identified a significant enrichment (<10^−4^) in W-box elements (Table S3 (Supporting Information)) which are known to bind WRKY TFs resulting in the activation of defense related gene transcription [80]. The web-based POBO program revealed that the invariant core W-box motif (TTGAC) was present in 50/51 genes at an average of 3.73 copies/promoter (t-test p-value <0.0001) while the stringent W-box motif (TTGAC(C/T)) was present in 43/51 genes at an average of 2.12 copies/promoter (t-test p-value <0.0001) (Table S3 (Supporting Information)). The *AtWAKL10* promoter contained two copies of the stringent and three copies of the core W-box motif. The presence of multiple copies of W-boxes in a high percentage of the promoters in the *AtWAKL10*-ECGG50 indicates that they may function as important regulatory elements in the gene group and thus WRKY TFs may be important in regulating the expression of the correlated genes.

## Discussion

The intracellular second messenger cGMP has been shown to be an important signaling molecule in biotic stress responses in plants however; to date no GC has been identified that is responsible for generating cGMP in response to pathogen challenge. Here we show that the intracellular domain of the AtWAKL10 protein that was previously shown to contain a potential GC motif [17] can generate cGMP *in vitro*. Further, we show that *AtWAKL10* is consistently co-expressed with genes that have well defined functional roles in early pathogen defense responses and additionally show that the expression of *AtWAKL10* and its correlated genes are induced early and markedly in response to a range of pathogens and their elicitors. This indicates that *AtWAKL10*, which encodes an experimentally confirmed GC protein, has a functional role in early defense responses making it an interesting candidate molecule that may be, at least in part, responsible for pathogen-induced cGMP generation.

Since cGMP has been shown to be generated and to function as an essential signaling molecule in defense against pathogens, we were particularly interested to investigate if one of our seven previously identified candidate GCs [17] may function in biotic defense responses. We selected AtWAKL10 since WAK family members have previously been implicated to function in these processes [24], [26]. Additionally, the structure of WAK proteins makes them likely candidates that may function in the recognition of extracellular elicitors and/or changes in the cell wall pectin composition since they contain extracellular EGF repeats that may function as ligand-binding domains and have been shown to bind tightly with cell wall pectin related compounds [23]. They could then transmit extracellular signals to the cytoplasm via their intracellular protein kinase domain [81] and/or, for AtWAKL10, the predicted GC domain that may signal to the cytoplasm by generating cGMP.

### *In Vitro* Enzymatic Activity of AtWAKL10_431–700_

The experiments presented here provide evidence that the intracellular domain of the AtWAKL10 protein can indeed function as a *bona fide* GC in Arabidopsis since the purified truncated recombinant AtWAKL10_431–700_ protein was shown to generate cGMP *in vitro* using two independent methodologies, an enzyme immunoassay and mass spectroscopic analysis (Figure 2B and 3A). Two other proteins identified using this GC search motif (or variations thereof) have previously been shown to generate cGMP *in vitro* [17] lending confidence to our search parameters designed to identify novel GCs in higher plants.

The maximum GC activity determined for AtWAKL10 of 150 fmoles cGMP generated/µg protein/15 min was markedly higher than values previously reported for AtBRI1 (approximately 65 fmoles/µg protein/15 min) [82] and AtGC1 (approximately 20 fmoles/µg protein/15 min) [17]. The greater GC activity of AtWAKL10 may reflect its increased importance as a cGMP generating molecule and/or that the *in vitro* assaying conditions were more suitable for AtWAKL10.

The ability of AtWAKL10_431–700_ to additionally function as a kinase indicates that the cytosolic portion of the AtWAKL10 molecule contains dual functional catalytic signaling domains. This is particularly noteworthy since the GC domain is located within the kinase domain and thus it is plausible that they may communicate via cross-talk (Figure 1B). It appears that GC domains tend to combine with other domains to make complex twin or multifunctional enzymes [83]. In *Chlamydomonas reinhardtii* >100 annotated nucleotide cyclases come in 22 different domain organizations with different catalytic partners including protein kinase-like domains, proteases and ATPase domains of HSP90 [83]. The best studied domain combinations in higher eukaryotes are GC and heme nitric oxide/oxygen-binding (H-NOX) [84] in sGCs and GC and kinases in the particulate rGCs [85]. The first is typical for sGCs, where binding of NO to the H-NOX domain is essential for activation of the GC. In the second type, the functional relationship, if any, between the GC and the kinase remains unknown.

### Computational Based Expression Analysis to Infer AtWAKLl0 Function

The strong induction of *AtWAKL10* expression in response to conditions that activate pathogen defense responses along with its consistent co-expression with genes that are known to have well established roles in pathogen defense responses strongly supports that AtWAKL10 may have a functional role in these processes. It is acknowledged that an increase in gene expression does not necessarily guarantee an increase in protein abundance and thus a functional response due to other modes of regulation such as post-transcriptional and -translational processing. However, transcription is the primary level of regulation in inducing protein expression since all other modes of regulation are dependent on a gene being initially transcribed. Thus, an increase in protein abundance is ultimately dependent on an increase in transcription.

The strong and rapid induction of *AtWAKL10* in response to a range of pathogens and their characteristic MAMPs including fungi (chitin), bacteria (elf26, flg22, HrpZ) and oomycetes (NPP1) provides increased resolution indicating that AtWAKL10 may function in early MAMP initiated basal defense responses to a broad range of pathogens [86]. MAMPs that are characteristic of different pathogens/microbes are detected on the surface of the PM by specific pattern recognition receptors that subsequently initiate convergent pathogen induced signaling and defense responses [87]. These responses include activation of defense related gene transcription and apoplastic defense [48], [49]. The co-expression of *AtWAKL10* with genes that encode PM localized proteins that function in pathogen recognition and signaling (*CERK1, BAK1*, lectin kinases and the *PROPEP* peptides and their receptors), and in apoplastic defense including (*SYP122*, *PEN1*, *VAMP722*, *SNAP33* and *PDR12),* indicates that AtWAKL10 functions in MAMP initiated basal defense responses. The *de novo* protein synthesis independent induction of the ECGG (in response to CHX) is consistent with them functioning as immediate early response genes [76] which play important roles in the early regulation of defense responses [77].

The induced expression of *AtWAKL10* by both biotrophic (*P. syringae*, *P. infestans*, *E. cichoracearum* and *G. orontii*) and nectrophic (*B. cinerea*, and OGs) pathogens and their related elicitors further indicates a role for AtWAKL10 against a broad range of pathogens. This is also supported by its consistent co-expression with genes involved in the synthesis of major pathogen defense signaling molecules including, SA (*EDS5/SID1, ICS1/SID2,* and *CBP60g*) and JA *(AOC3*, *OPR3* and *LOX3),* and those involved in the biosynthesis of the antimicrobial phytoalexin camalexin (*PAD3*), since these molecules are involved in defense against both biotrophic and nectrophic pathogens. While SA and JA are typically involved in defense against biotrophic and nectrophic pathogens respectively [88], camalexin is synthesized early and locally in an SA- and JA-dependent manner in response to a broad range of biotrophic and nectrophic pathogens [89].

Also linking AtWAKL10 to early defense responses is the presence of a number of calcium transporting and sensing molecules in the ECGG50 since calcium is an important second messenger in plant defense and rapid increases in cytosolic calcium concentrations are required for activation of downstream defense signaling following pathogen recognition (Table S1) [56]. Significantly, a number of the early defense related co-expressed genes encode proteins whose activities are known to be regulated by calcium including the MLO proteins [90], SYP122 [49] and CBP60g [56].

It is particularly relevant to note here that the AtWAKL10 protein has a predicted extracellular calcium-binding EGF-like domain [18] since pathogen-induced increases in cytosolic calcium are mirrored by a reduction in apoplast calcium concentrations which have been suggested to influence cell wall rigidity due to the role of calcium in non-covalent cell wall cross-linking [91]. In addition, the extracellular domain of AtWAKL10 contains a similar composition of basic aas and conserved cysteine residues that were determined and suggested, respectively, to be responsible for AtWAK1 PGA (homogalacturonan) binding [29]. This, combined with the strong and early induction of *AtWAKL10* expression following OG challenge, makes it plausible that AtWAKL10 may detect changes in apoplastic calcium concentrations and cell wall pectin composition and signal to the cytoplasm via its intracellular kinase and/or GC domain.

The enrichment of WRKY TF binding sites (TFBS, W-boxes, TTGAC-core and TTGACC/T-stringent) in the promoters of the *AtWAKL10-*ECGG50, including the promoter of *AtWAKL10* (Table S3 (Supporting Information)), is also consistent with their induced expression in response to pathogens since WRKY TFs have a well documented role in regulating the expression of defense response genes [92]. In eukaryotes, it has been documented that genes that are determined to have highly correlated expression values (*r*>0.84) over a large number of diverse experiments, such as those determined here, are likely to contain common TFBS in their promoters and thus be co-regulated [37]. In addition, two WRKY TFs (−28 and −15) were found to be co-expressed with *AtWAKL10* although these have not been shown to be involved in regulating the expression of biotic stress related genes.

In summary, the correlation analysis revealed that *AtWAKL10* is consistently co-expressed with a number of genes that are known to function in early pre- and post-invasive defense responses to pathogens including receptor linked protein kinases, PM proteins, calcium transporting and signaling molecules, cell wall strengthening, phytoalexin synthesis and genes involved in the biosynthesis and regulation of the main defense signaling molecules including SA and JA. The stimulus-specific expression analysis complementing this analysis revealed that expression of *AtWAKL10* and the ECGG50 is strongly and largely uniformly induced early in response to a broad range of pathogens and their elicitors.

The ability of the intracellular domain of AtWAKL10 to generate cGMP *in vitro* combined with its inferred role in early pathogen defense responses is of particular interest since cGMP levels have been shown to increase in response to pathogen challenge in Arabidopsis [12] and cGMP been shown to be an essential signaling molecule in pathogen defense responses in plants [5]. Further, cGMP has also been shown to be required for the induced expression of the defense-related genes *PAL* and *PR-1* and also for activation of PAL enzyme activity which is a requirement for SA biosynthesis in tobacco [5]. Evidence indicates that cGMP triggers increases in cytosolic calcium levels that in turn activate SA biosynthesis and accumulation resulting in the activation of downstream SA signaling pathways [14]. The early pathogen-induced induction of *AtWAKL10* and its confirmed co-expression with key genes involved in SA biosynthesis in Arabidopsis, including *EDS5* and *ICS1*, as well as calcium sensing molecules corresponds well with previous studies that link cGMP to calcium and SA biosynthesis. It is conceivable that expression of a cGMP-generating signaling molecule such as *AtWAKL10* is part of the response to pathogen challenge to meet the increased requirement for cGMP production.

## Materials and Methods

### AtWAKL10 Structure and Sequence Comparison

The protein sequences of AtWAKL10 and AtWAK1 were analyzed for the presence of known domains and functional sites using the PROSITE database located within the Expert Protein Analysis System (ExPASy) proteomics server (http://www.expasy.ch/). Additionally, the aa sequences of AtWAKL10 (Q8VYA3) and AtWAK1 (Q39191) were obtained from ExPASy and their similarities were compared using the EMBOSS Pairwise Alignment Algorithm (http://www.ebi.ac.uk/Tools/emboss/align/index.html).

### AtWAKL10 Recombinant Protein

#### Cloning, expression and purification of AtWAKL10

Total RNA was extracted from three week old Arabidopsis ecotype Columbia-0 (Col-0) seedlings using the RNeasy plant mini kit, in combination with DNase 1 treatment, as instructed by the manufacturer (Qiagen, Crawley, UK). *AtWAKL10* cDNA was synthesized from total RNA using Superscript III reverse transcriptase (Invitrogen, Paisley, UK) and subsequent PCR amplification performed with the appropriate primers (forward: 5′-GGGTCGACGATATTAATGAATGCGTA-3′ incorporating a *SalI* restriction site; reverse: 5′-GCCTCGAGTACTTGTCTCATACTTGG-3′ incorporating an *XhoI* restriction site) to transcribe the gene region that encodes aas _431–700_. The PCR product was then cloned into the pCRT7/NT-TOPO expression vector (Invitrogen, Carlsbad, USA) to make a pCRT7/NT-TOPO-AtWAKL10 fusion expression construct with an N-terminal His purification tag. The construct was maintained in TOP10 F′ *Escherichia coli* cells (Invitrogen, Carlsbad, USA).

For expression of the recombinant protein AtWAKL10_431–700_, *E. coli* BL21 Star pLysS cells (Invitrogen, Carlsbad, USA) were transformed with the pCRT7/NT-TOPO-AtWAKL10 construct and grown in double strength yeast-tryptone media (16 g/L tryptone, 10 g/L yeast extract, 5 g/L NaCl and 4 g/L glucose; pH 7.0) containing 100 µg/ml ampicillin and 34 µg/ml chloramphenicol, on an orbital shaker at 37°C. Expression of the truncated recombinant AtWAKL10_431–700_ protein was induced when the optical density (OD_600_) of the cell culture had reached 0.6 (approximately 3 h), by the addition of isopropyl-β-D-thiogalactopyranoside (IPTG) to a final concentration of 1 mM and the culture left to grow for a further 3 h at 37°C.

The recombinant AtWAKL10_431–700_ was purified by preparing a cleared cell lysate of the induced *E. coli* cells under denaturing conditions where the harvested cells were resuspended in lysis buffer (8 M urea, 100 mM NaH_2_PO_4_, 10 mM Tris-Cl; pH 8.0, 500 mM NaCl, 20 mM β-mercaptoethanol and 7.5% v/v glycerol) at a ratio of 1 g pellet weight to 10 mL buffer volume, mixed thoroughly using a mechanical stirrer at 24°C for 1 h and then centrifuged at 2300×*g* for 15 min. The supernatant was collected as the cleared lysate and transferred to 2 mL of 50% Ni-NTA slurry (Qiagen, Crawley, UK) that had been pre-equilibrated with 10 mL of lysis buffer and then gently mixed on a rotary mixer for 1 h at 24°C. The lysate-resin mixture was loaded into an empty XK16 column (Amersham Pharmacia Biotech, Little Chalfont, UK), allowed to settle and the flow-through discarded. The resin was washed three times with 30 mL wash buffer (8 M urea, 100 mM NaH_2_PO_4_, 10 mM Tris-Cl; pH 8.0, 500 mM NaCl, 20 mM β-mercaptoethanol, 7.5% v/v glycerol and 40 mM imidazole). Subsequently, the resins were equilibrated with 2 mL gradient buffer (8 M urea, 200 mM NaCl, 50 mM Tris-Cl; pH 8.0 and 20 mM -mercaptoethanol) before the column was connected to an AKTA fast protein liquid chromatography (FPLC) (Amersham Pharmacia Biotech, Little Chalfont, UK) programmed to run a linear refolding gradient. The refolding gradient for the denatured recombinant AtWAKL10 was performed by linearly diluting the 8 M gradient buffer to a 0 M urea concentration with a refolding buffer (200 mM NaCl, 50 mM Tris-Cl; pH 8.0, 500 mM glucose, 0.05% w/v poly-ethyl glycol, 4 mM reduced glutathione, 0.04 mM oxidized glutathione, 100 mM non-detergent sulfobetaine and 0.5 mM phenylmethanesulfonylfluoride (PMSF)) over 90 min. After renaturation, the recombinant protein was eluted in 2 mL of elution buffer (200 mM NaCl, 50 mM Tris-Cl; pH 8.0, 250 mM imidazole, 20% v/v glycerol and 0.5 mM PMSF). The eluted protein fraction was then de-salted and concentrated using centriplus filtration devices with molecular weight cut-off point of 3000 Da, according to the manufacturer's instructions (Millipore Corporation, Bedford, USA). Protein concentration was determined by the Bradford method [93]. The predicted mass of the truncated AtWAKL10_431–700_ protein was determined using the ProtParam tool in the ExPASy Proteomics Server (http://au.expasy.org/tools/protparam.html). The resulting purified AtWAKL10_431–700_ protein was tested for GC and kinase activity.

#### Cyclic nucleotide assays

The GC activity of the purified AtWAKL10_431–700_ protein was measured *in vitro* by incubating 10 µg of protein in 50 mM Tris-Cl; pH 8.0, 2 mM isobutylmethylxanthine (IBMX) (phosphodiesterase inhibitor), 5 mM Mg^2+^ and/or 5 mM Mn^2+^ and 1 mM GTP, in a final volume of 100 µl [94]. Background cGMP levels were measured in tubes that contained the incubation mediums but no protein. Incubations were performed for 5, 10, 15 and 20 min at room temperature (24°C) and terminated by the addition of 10 mM EDTA. Tubes were then boiled for 3 min, cooled on ice for 2 min and centrifuged at 2300×*g* for 3 min. The resulting supernatant was assayed for cGMP content using the cGMP enzyme immunoassay Biotrak (EIA) System following the acetylation protocol described in the supplier's manual (Amersham Pharmacia Biotech, Little Chalfont, UK; code RPN226). The anti-cGMP antibody is highly specific for cGMP and has approximately 106 times lower affinity for cAMP. In order verify and validate the result we obtained with this antibody based detection method, we also used mass spectrometry, a method capable of specifically and sensitively detecting cGMP at fmol concentrations. Each experiment was performed in triplicate (n = 3).

Mass spectroscopic determinations of cGMP generated by recombinant AtWAKL10_431–700_ *in vitro* were done with a Waters API Q-TOF Ultima (Waters Microsep, Johannesburg, South Africa) in the W-mode. The samples were introduced with a Waters Acquity UPLC (Waters Microsep, Johannesburg, South Africa) at a flow rate of 180 µL/min and separation was achieved by a Phenomenex Synergi 4 µm Fusion-RP (250×2.0 mm) column (Torrance, CA, USA). A gradient of solvent “A” (0.1% v/v formic acid) and solvent “B” (100% acetonitrile) over 18 min was applied. During the first 7 min the solvent composition was kept at 100% “A” followed by a linear gradient over 3 min to 80% “B” and re-equilibration to the initial conditions. Electrospray ionisation in the negative mode was used at a cone voltage of 35 V. The running parameters were optimized for sensitivity and specificity.

Substrate specificity of AtWAKL10 for GTP was also assessed by testing the ability of the recombinant to generate cAMP from ATP. A reaction mixture containing 10 µg of the purified recombinant AtWAKL10_431–700_, 50 mM Tris-Cl; pH 8.0, 1 mM ATP, 2 mM IBMX, and 5 mM Mg^2+^ was prepared in a final volume of 100 µl and incubated for 15 min at room temperature. The reaction was terminated as described previously and the cAMP product retrieved by centrifugation and measured by mass spectrometry. Each experiment was performed in triplicate (n = 3).

#### Kinase assay

The kinase activity of the purified recombinant AtWAKL10_431–700_ protein was assessed *in vitro* by measuring its ability to direct the phosphorylation of a special Ser/Thr substrate peptide as described in the Omnia™Ser/Thr-Recombinant Kit (KNZ2011, BioSource, Nivelles, Belgium). A 50 µl reaction mix containing 0.1 ng purified recombinant AtWAKL10_431–700_, 1 x reaction buffer, 1 mM ATP or GTP, 0.2 mM DTT, and 25 µM Ser/Thr-peptide was prepared. The mix was then excited at a wavelength of 360 nm and fluorescence measured at the emission wavelength of 460 nm every min for 20 min at 30°C. All activity readings were recorded as relative fluorescence units (RFUs) on a Modulus Microplate Reader (Turner BioSystems, Sunnyvale, CA, USA). Further, the effects of 1 µM cGMP on the kinase activity of the recombinant AtWAKL10 were also assessed using the same assaying system. Each experiment was performed in triplicate (n = 3).

### Computational Based Analysis

#### AtWAKL10 co-expression analysis

The Arabidopsis co-expression tool (ACT) (http://www.arabidopsis.leeds.ac.uk) [95] was used to perform the correlation analysis using *AtWAKL10* (At1g79680; Probe ID: 261394_at) as the driver gene. The analysis was performed across all available experiments leaving the gene list limit blank to obtain a full correlation list.

#### Functional enrichment of the *AtWAKL10*-ECGG50

The “Fatigoplus” (version 3.1) compare tool in the Babelomics suite (http://babelomics.bioinfo.cipf.es) [40] was used to identify any significant enrichments in functional terms associated with the *AtWAKL10-*ECGG50. All available Arabidopsis data bases were selected using default options which included; GO (BP, MF and CC, annotation levels 3–9), KEGG pathways and Swissprot keywords. Significance is determined using an adjusted p-value to correct for multiple hypothesis testing.

#### Stimulus-specific microarray expression profiling

The expression profiles of the *AtWAKL10-*ECGG50 were initially screened over all of the available ATH1: 22K array Affymetrix public microarray data in Genevestigator V3 (https://www.genevestigator.com) using the stimulus and mutation tools [96]. In order to obtain greater resolution of gene expression profiles, the normalized microarray data were subsequently downloaded and analyzed for experiments that were found to induce differential expression of the genes. The data were downloaded from the following repository sites; NASCArrays (http://affymetrix.arabidopsis.info/narrays/experimentbrowse.pl) [97], TAIR-ATGenExpress (http://www.ebi.ac.uk/microarray-as/ae/) and GEO (NCBI) (http://www.ncbi.nlm.nih.gov/geo/) [98] (see Text S2 (Supporting Information)). The array data were analyzed and fold change (log2) values were calculated for each experiment. Expression heat maps were generated using the Multiple Array Viewer program from the MultiExperiment Viewer (MeV) software package (vesion 4.2.01) created by The Institute for Genomic Research (TIGR) [99]. In order to confirm the microarray expression results for selected experiments, semi-quantitative RT-PCR analysis was performed for *AtWAKL10*.

#### Promoter analyses of the *AtWAKL10*-ECGG50

The promoter regions of *AtWAKL10* and the *AtWAKL10*-ECGG50 were analyzed for any enrichments in potential TFBS using the web-based Athena (http://www.bioinformatics2.wsu.edu/cgi-bin/Athena) [78] and POBO (http://ekhidna.biocenter.helsinki.ft/poxo/pobo) [79] applications. The visualization tool in Athena performs an analysis of Arabidopsis promoter sequences and reports enrichments of known plant TFBS. The analysis of *AtWAKL10*-ECGG50 was performed using settings of 1000 bp upstream of the transcription start site (TSS) and do not cut off at adjacent genes.

The Athena results were subsequently confirmed in POBO by uploading promoter sequences 1 kb upstream of the coding regions of the *AtWAKL10-*ECGG50. The analysis was run against Arabidopsis background (clean) searching for the stringent (TTGACC/T) and core (TTGAC) W-box motif using default settings. A two-tailed p-value was calculated in the linked online GraphPad web-site using the generated t-value and degrees of freedom to determine the statistical differences between input sequences and background.

### Plant Infections with *Pseudomonas syringae* or *Botrytis cinerea*

Virulent *Pst* [100] and avirulent *Pst avrB* [101] strains (supplied by Gail Preston, Department of Plant Sciences, Oxford University) were grown on Kings Broth (KB) [102] agar (1.5% w/v) supplemented with 50 µg/ml rifampicin for *Pst*; and 50 µg/ml rifampicin plus 50 µg/ml kanamycin for *Pst avrB* selection; for two days at 28°C. Bacterial inoculums were prepared and the leaves of four week old Arabidopsis Col-0 plants were infected with virulent *Pst* and avirulent *Pst avrB* using a previously described pressure infiltration protocol [103]. Briefly, leaves were inoculated with 1×10^5^ colony forming units (cfu)/mL *Pst* or *Pst avrB* suspended in 10 mM MgCl_2_ or with10 mM MgCl_2_ (control) and samples harvested at 1, 3, 6, 12, 24 and 48 hat.

The *B. cinerea* GLUK-1 (pepper) isolate [104] (supplied by Dr Gary Loake, University of Edinburgh) was maintained on sugar free apricot halves (Natuurlite, South Africa) at 25°C in the dark. Spores were harvested 14 days after subculture by adding 3 mL of sterile dH_2_O to the Petri dish containing the infected apricot and then gently rubbing the spores with a sterile glass rod until the dH_2_O appeared cloudy. The concentration of spores was determined using a haemocytometer and adjusted to 5×10^4^ spores/mL in half strength (v/v) grape juice (Liquifruit, RSA). Leaves of four week old Arabidopsis Col-0 plants were detached and placed onto 0.8% (w/v) agar plates. The upper leaf surface was inoculated with 5 µl of the spore suspension or half strength grape juice (control). Samples were then harvested 1, 3, 6, 12, 24 and 48 hat.

### Chemical Treatments of Plants and Transcript Analyses

Leaves of four week old Arabidopsis Col-0 plants were removed and placed in six well microtitre plates. Leaves were floated in 1 mM SA solution or 0.5% (v/v) ethanol (EtOH) (control) for 30 min after which time the leaves were removed, quickly patted dry and then placed into fresh microtitre plates in dH_2_O for the remaining time of treatment. Samples were harvested 1, 6, 12, 24 and 48 hat. For the CHX treatments, two week old Arabidopsis Col-0 seedlings were treated for 3 h with 10 µM CHX or the equivalent concentration (0.003%) of DMSO (control, C).

RNA was extracted using an adapted LiCl precipitation method [105]. The cDNA was reverse transcribed from 2.5 µg of total RNA using Superscript III reverse transcriptase according to the manufacturer's instructions (Invitrogen, Paisley, UK). Semi-quantitative RT-PCR analysis was then performed using a Gene Amp PCR System (Applied Biosystems, Foster City, USA). The ubiquitin conjugating enzyme E2 (*UBQ*) (At5g25760) (forward primer 5′-GGACCGCTCTTATCAAAGGA-3′and reverse primer 5′-CTTGAGGAGGTTGCAAAGGA-3′) was used as a “housekeeping gene” control. Twenty five PCR cycles of 94°C×15 sec, 65°C×30 sec and 72°C×1 min ensured product formation was semi-quantitative. *AtWAKL10* (forward primer 5′-AGGGAAGGAAACGACCAAGT-3′ and reverse primer 5′-GCGACGAAGATGTTGTAGCA-3′) product formation was semi-quantitative when 32 PCR cycles of 94°C×15 sec, 65°C×30 sec and 72°C×1 min was performed.

## Supporting Information

Table S1 Extended list of top 100 Arabidopsis genes co-expressed with AtWAKL10 (At1G79680).
(0.06 MB XLS)

Table S2 Fatigo GO analysis.
(0.04 MB DOC)

Table S3 Promoter analysis.
(0.06 MB DOC)

Text S1 Description of expression correlated pathogen defence related genes.
(0.22 MB DOC)

Text S2 Description of microarray experiments.
(0.03 MB DOC)
