# Supplementary material for: The Arabidopsis Wall Associated Kinase-Like 10 Gene Encodes a Functional Guanylyl Cyclase and Is Co-Expressed with Pathogen Defense Related Genes
Source: PLoS One. 2010 Jan 26;5(1):e8904. doi: 10.1371/journal.pone.0008904 (PMC2811198; doi:10.1371/journal.pone.0008904)
Supplement: Table S2 — Fatigo GO analysis. (0.04 MB DOC) [file pone.0008904.s002.doc]

**TABLE S2**

**FatiGO+ analysis**

**Gene Ontology :**

Summary input data:

Genes in list 1 (WAKL10-ECGG50): 51

Genes in list 2 (entire genome): 28755

| GO term | **WAKL10-ECGG50** | | | genome | |  | |
| --- | --- | --- | --- | --- | --- | --- | --- |
|  | **No. genes** | **%** | **No. genes** | | **%** | | **Adj.**  **p value** |
| **Biological Process** |  |  |  | |  | |  |
| defense response (L3) | 11 (39) | 28.21 | 604 (14455) | | 4.18 | | 2.50E-05 |
| response to biotic stimulus (L3) | 9 (39) | 23.08 | 489 (14455) | | 3.38 | | 1.57E-04 |
| response to other organism (L4) | 8 (34) | 23.53 | 446 (13863) | | 3.22 | | 1.51E-03 |
|  |  |  |  | |  | |  |
| **Cellular Component** |  |  |  | |  | |  |
| plasma membrane (L5) | 5 (30) | 16.67 | 378 (12221) | | 3.09 | | 2.61E ^-02^ |
|  |  |  |  | |  | |  |

**L =** level of GO category

**No. genes** = number of genes in the specified list with the annotated GO term. The numbers in brackets (X) indicates the total number of genes in each list that are annotated at the indicated GO category and level.

**%** = percentage of annotated genes at the indicated category that contain the defined GO term

**Adjusted p-value** = Family Wise Error Rate (FWER).

**Enriched genes identified in the GO analysis**

**Biological Process**

defense response:

AT4G11170, AT3G25780, AT4G39030, AT1G57630, AT3G52400, AT1G66090, AT2G35980, AT1G61560, AT1G57650, AT2G32140, AT4G33430

response to biotic stimulus:

AT3G25780, AT4G39030, AT3G52400, AT2G35980, AT1G61560, AT3G26830, AT1G15520, AT4G33430, AT5G05730

response to other organism:

AT3G52400, AT1G61560, AT1G29690

**Cellular Component**

plasma membrane:

AT4G33430, AT3G25780, AT3G52400, AT1G61560, AT1G15520
