# Supplementary material for: The Arabidopsis Wall Associated Kinase-Like 10 Gene Encodes a Functional Guanylyl Cyclase and Is Co-Expressed with Pathogen Defense Related Genes
Source: PLoS One. 2010 Jan 26;5(1):e8904. doi: 10.1371/journal.pone.0008904 (PMC2811198; doi:10.1371/journal.pone.0008904)
Supplement: Table S3 — Promoter analysis. (0.06 MB DOC) [file pone.0008904.s003.doc]

**TABLE S3**

**PROMOTER ANALYSIS**

**A: ATHENA promoter analysis**

| **Search sequence** | **No. promoters searched** | **No. promoters with site** | **Total No. sites** | **Av. copies /promoter** | **P-value** |
| --- | --- | --- | --- | --- | --- |
| **TTGAC(A/T)** | 51 | 44 (86.3%) | 103 | 2.02 | < 10 ^- 4^ |

The W-box sequence **TTGAC[C/T]** is enriched in these genes with a corrected P-value of <10^-4^.

**B: POBO analysis results summary**

# SETTINGS

Search pattern = TTGAC[C/T] and TTGAC

Background (BG) model: *Arabidopsis_thaliana*_clean (all promoters)

number of sequences to pick-out = 50 (default)

number of samples (pseudoclusters) to generate = 1000 (default)

sequence length = 1000 bps

Cluster 1 input = promoter sequences (1kb upstream coding region) from WAKL10 ECGG50

**RESULTS**


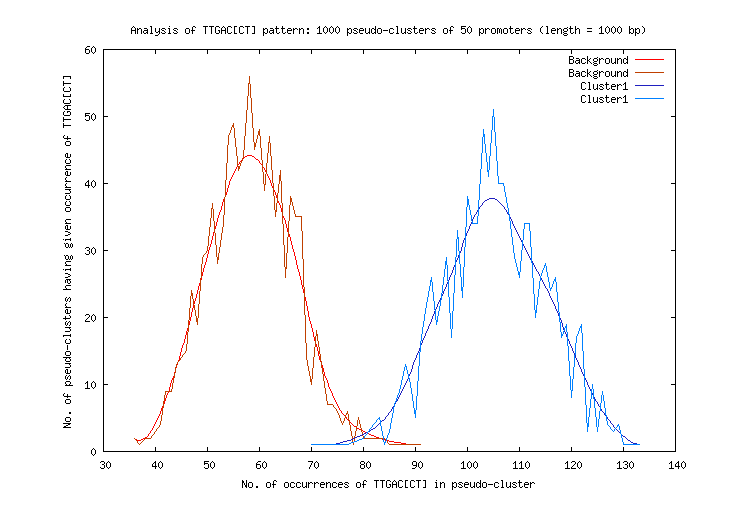

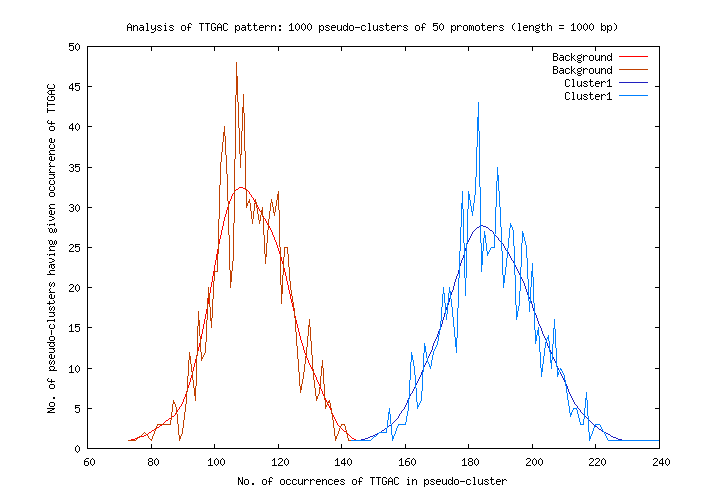


Illustration of the frequency of the occurrence of the search patterns (TTGAC[C/T] and TTGAC) in the artificial clusters generated from the background and input sequences.

**POBO SUMMARY TABLE**

| **Motif** | **Data set** | **No. prom. in dataset** | **No. prom**  **with motif** | **Total No. motif in dataset** | **Av.copies /**  **promoter** |
| --- | --- | --- | --- | --- | --- |
| **TTGAC[C/T]** | WAKL10 | 1 | 1 | 2 | 2 |
|  | BG | 31353 | 20911 (87.8%) | 36752 | 1.16 |
|  | ECGG50 | 51 | 50 (98.0%) | 190 | 2.12 |
|  |  |  |  |  |  |
| **TTGAC** | WAKL10 | 1 | 1 | 3 | 3 |
|  | BG | 31353 | 27523 (87.8%) | 70245 | 2.25 |
|  | ECGG50 | 51 | 50 (98.0%) | 190 | 3.37 |

**STATISTICS**

Independent T-test between Cluster 1 and background (TTGAC[C/T])

t-value = 113.62, Degrees of Freedom =1998.

Independent T-test between Cluster 1 and background (TTGAC)

t-value = 126.5, Degrees of Freedom =1998

The two-tailed P value (calculated using the linked online GraphPad web-site; http://www.graphpad.com/quickcalcs/DistMenu.cfm) was determined to be less than 0.0001 for both motifs indicating that the enrichment in the WAKL10-ECGG50 is extremely statistically significant.

**Location of W-box motifs in WAKL10 promoter (upstream of coding region)**

| **Motif** | **start** | **end** | **strand** | **pattern** |
| --- | --- | --- | --- | --- |
| **TTGAC[C/T]** | -712 | -706 | + | TTGACC |
|  | -98 | -92 | - | GGTCAA |
|  |  |  |  |  |
| **TTGAC** | -712 | -707 | + | TTGAC |
|  | -669 | -664 | + | TTGAC |
|  | -97 | -92 | - | GTCAA |
