# Supplementary material for: The Arabidopsis Wall Associated Kinase-Like 10 Gene Encodes a Functional Guanylyl Cyclase and Is Co-Expressed with Pathogen Defense Related Genes
Source: PLoS One. 2010 Jan 26;5(1):e8904. doi: 10.1371/journal.pone.0008904 (PMC2811198; doi:10.1371/journal.pone.0008904)
Supplement: Text S1 — Description of expression correlated pathogen defence related genes. (0.22 MB DOC) [file pone.0008904.s004.doc]

**Text S1**

**Description of expression correlated pathogen defence related genes**.

The plasma membrane (PM) localised SYntaxin of Plants (SYP)-122 (r = 0.828, At3G52400) functions in vesicle-membrane fusion events and is the most closely related gene and shares functional redundancy with the Penetration (PEN)-1 syntaxin (SYP121, r = 0.695, At3G11820) which has an essential functional role in resistance to *Blumeria graminis* f. sp. *hordei* penetration [1] [2] [3]. Transcription of *SYP122* has been shown to be induced in response to a range of pathogens and the SYP122 protein has been shown to be phosphorylated in a calcium dependent manner in response to bacterial flagellin elicitation [2]. Along with PEN1, the SYP122 protein has been shown to accumulate in the PM at fungal attack sites and it appears to have a general secretory function that includes the secretion of proteins into the cell wall [1] [4]. In addition to these syntaxins, *AtWAKL10* expression is also correlated with genes encoding other essential proteins of the SNARE (Soluble-NSF (N-ethylmaleimide-sensitive factor) protein Attachment protein Receptor) machinery including *synaptosomal-associated protein (SNAP)-33* (r = 0.722, At5G61210) and *Vesicle-Associated Membrane Protein (VAMP)-722* (r = 0.734, At2G33120) which function in the directed exocytosis of vesicle contents that can contain defense-related proteins and compounds [2] [5].

The PM localised pleiotropic drug resistance (PDR)-12 / adenosine 5’-triphosphate (ATP)-Binding Cassette (ABC) transporter (r = 0.866 At1G15520) has been placed in the same PDR subfamily as the PEN3 ABC transporter (r =0.558, PDR8) [6]. The ABC transporters function as efflux pumps involved in the focal delivery of antimicrobials, including PEN2-generated toxic compounds, and/or agents that promote chemical cross-linking of plant cell wall polymers, into the apoplastic space [7] [8]. Expression of *PDR12* has been shown to be induced by biotic and abiotic stresses, SA, ETH and JA and is thought to be involved in transporting antimicrobial secondary metabolites to the cell surface [6,9]. The expression of *PDR12* was dramatically enhanced in *pen3* mutant plants challenged with *B. g. hordei* which may suggest functional redundancy [6].

The PM localized *MILDEW RESISTANCE LOCUS (MLO)-6* (r = 0.865, At1G61560, position 11) is a co-ortholog of the barley MLO gene that functions in early fungal-host interactions [10]. Transcription of *MLO6* has previously been reported to be up regulated in response to both biotrophic and nectrophic pathogens and abiotic stresses [11]and along with the MLO2 (r = 0.511) and MLO12 (r = 0.730) co-orthologs have a partial functional redundancy in the establishment of compatibility with biotrophic powdery mildew species including *G. orontii* and *E. cichoracearum* but function in resistance against necrotrophic (*Alternaria spp.*) and hemibiotrophic (*P. infestans*) pathogens indicating that MLO proteins mediate host responses to diverse pathogen species [12]. MLO proteins have been shown to co-localise with syntaxins at the PM beneath attempted fungal penetration sites and it has been suggested that they may modulate SNARE protein dependent delivery of antimicrobial containing vesicles [13] [10].

The PM localized Brassinosteroid insensitive Associated receptor Kinase 1 (BAK1, r = 0.810, At4G33430) protein has been shown to rapidly form complexes with the flagellin-sensing 2 receptor (FLS2; r =0.416) after flagellin stimulation [14] and be an essential signalling component in bacterial flagellin and EF-Tu-triggered immune responses [15]. It has been suggested that BAK1 may function as a convergent point in MAMP signalling and integrate signals from diverse MAMP receptors and mediate appropriate downsteam responses to a diverse range of microbes [14] [16]. The *BAK1* gene has also been implicated to function in defence responses against *B. cinerea* since it is required to contain pathogen induced cell death and necrosis which results in enhanced susceptibility to *B. cinerea* [17].

The *PROPEP2* (r= 0.844, At5g64890) and *PROPEP3* (r = 0.708, At5g64905) paralog genes which encode peptides that function as endogenous amplifiers of innate immune defence responses through a MAMP initiated feedback loop which amplifies signalling through both the JA/Et and SA pathways [18]. The expression of *PROPEP2 and -3* is strongly induced in response to SA, JA (*PROPEP2* only), fungal, bacterial, and oomycete pathogens and their associated MAMP elicitors [19]. The encoded proteins are secreted into the apoplast where they can specifically interact with the co-expressed cell-surface leucine-rich repeat (LRR) receptor kinase, PEP1 RECEPTOR (PEPR)-1 (r = 0.800, At1G73080) and amplify defence signaling [18]. Further, WAKL10 was also correlated with the *PEPR2* gene (r = 0.777, At1G17750) which encodes a protein that is similar to *PEPR1* [20].

Five disease resistance proteins are annotated to be involved in defence responses and contain domains that are characteristic of R-proteins, including; nucleotide binding site (NBS), leucine rich repeats (LRR) and Toll-Interleukin receptor domains (TIR) [21]. The R-proteins typically detect specific pathogen secreted effectors and activate effector triggered immune (ETI) responses [22].

The expression of *AtWAKL10* is also highly correlated with genes involved in the biosynthesis of the major pathogen defence signalling molecules SA and JA (Glazebrook 2005).

The *Salicylic acid Induction Deficient (SID)-1/ENHANCED DISEASE SUSCEPTIBILITY (EDS)-5* (r = 0.841, At4G39030 (r = 0.841, At4G39030) [23] and *SID-2/IsoChorismate Synthase (ICS)-1* (r = 0.810, At1G74710) [24] genes are both critically involved in the biosynthesis SA while *calmodulin-binding protein* (*CBP)-60g* (r = 0.825, At5G26920) has recently been shown to be induced by MAMPs and *P. syringae* and mutation studies have indicated that CBP60g is required for limiting pathogen growth and for normal pathogen induced SA accumulation [25]. In contrast, the co-expressed *Constitutively Active cell Death (CAD)-1* (r = 0.841, At1G29690) gene has been shown to be induced rapidly in response to chitin elicitation (20min) and SA (1h) and mutant studies indicate that the CAD1 protein is a negative regulator of endogenous SA levels and SA-mediated programmed cell death immunity [26] [27]. Correlated genes involved the biosynthesis of JA include *Allene Oxide Cyclase (AOC)-3* (r = 0.830, At3G25780), *oxophytodienoate reductase (OPR)-3* (r = 0.747, At2G06050) and *lipoxygenase (LOX)-3* (r = 0.704, At1G17420) [28].

While it is generally accepted that SA and JA activate mutually antagonistic defence signalling pathways in order to induce appropriate defense responses against biotrophic and nectrophic pathogens respectively, it has been shown that a complex cross-talk occurs between these pathways that also involve synergistic interactions in order to fine tune appropriate defense responses [29,30].

*AtWAKL10* is also consistently co-expressed with genes encoding enzymes that function in the biosynthesis of tryptophan and camalexin which is the main antimicrobial phytoalexin in Arabidopsis that is synthesised in response to a broad range of biotrophic and nectrophic pathogens [31] [32]. These genes include *Anthranilate Synthase Alpha (ASA)-1* (r = 0.819, At5G05730), *Tryptophan Synthase Alpha (TSA)-1* (r = 0.731, At3g54640), and *Indole-3-Glycerol Phosphate Synthase* (*IGPS*; r=0.799, At2G04400) that are involved in tryptophan biosynthesis and *PhytoAlexin Deficient (PAD)-3* (r = 0.859, At3G26830) that encodes the final enzyme required for the biosynthesis of camalexin [31] [33] [34] [35] [36]. Camalexin biosynthesis has been shown to occur at localized sites of pathogen infection [37] and its formation is coupled to induction of *ASA1* and *TSA* expression [33] [36].

Other interesting correlated genes that may function in pathogen defence responses include the two most highly correlated genes which are both lectin protein kinases (At5G65600, r = 0.914 and At4G21390, r =0.907). In plants, lectins play important roles in stress responses and defence and are considered one of the most important recognition molecules that primarily interact with foreign glycans such as chitin [38] [39].

A number of correlated genes have also been implicated to function in regulating the transcription of defence related genes including two WRKY family transcription factors (TFs) (*WRKY28*, r = 0.820, At4G18170; and *WRKY15*, r = 0.790, At2G23320) [40]. Expression of the *ETHYLENE RESPONSE FACTOR (ERF)-1A* TF (r = 0.845, At4G17500) has been shown to be induced early in response to pathogens and their elicitors, CHX, SA, JA and ethylene [41,42] [43] and activates transcription of genes that contain GCC boxes (AGCCGCC) in their promoters which have been associated with the defense transcriptome including several *PATHOGENESIS-RELATED (PR)* genes [43] [41]. The *Jasmonate-ZIM-DOMAIN PROTEIN (JAZ)-1* (r = 0.862, At1G19180) gene encodes a negative regulator of the MYC2 TF [44] [45] which differentially regulates two classes of JA regulated genes [46] and is a negative regulator of defense genes that function in resistance to *B .cinerea* including those involved in tryptophan metabolism [46].

Based on amino acid similarity [47], the Yellow Leaf Specific 9 (YLS9) / Non-race specific Disease Resistance-1 (**N**DR1) / (Hairpin-Induced-1 (**H**IN1) -**L**ike 10 (NHL10) (r = 0.868) is thought to be the *A. thaliana* ortholog of the tobacco HIN1 gene whose expression is rapidly induced by a proteinous elicitor secreted by plant bacterial pathogens [48]. NHL10 and HIN1 have common expression characteristics in that their transcription is induced during the hypersensitive response (HR) caused by exposure to an avirulent Cucumber mosaic virus (CMV) strain and by spermine, which is a novel polyamine HR defence signaling molecule and an inducer of tobacco PR genes[49] and their transcripts are abundant in senescing leaves [47]. The induction of HIN1 by AvrPto in tobacco has been shown to be dependent on an in-tact HR and Pathogen (Hrp) secretion system [48]. It was shown that while the senescence-associated induction of NHL10 is SA-dependent, the induction observed in response to CMV-induced HR and Spm application occurred independent of SA [47].

A number of calcium transporting and sensing molecules are also highly correlated with WAKL10 including a PM calcium-transporting ATPase (r = 0.856, At3G63380), the calmodulin-binding protein (CBP)-60g (r = 0.824, At5G26920), a calmodulin binding phospholipid-transporting ATPase 10, that may be involved in the transmembrane movement of ions including calcium (r = 0.794, At3G25610), a calcium ion binding molecule that is similar to calmodulin-like (CML)-38 (r = 0.783, At3G01830) and calcium-dependent protein kinase (CDPK)-10 ( r = 0.776, At1G18890). This is interesting because rapid increases in cytosolic calcium concentrations have shown to occur and be required for activation of downstream defense signaling following pathogen recognition [50] [51] [52] [25].

REFERENCES

1. Assaad FF, Qiu JL, Youngs H, Ehrhardt D, Zimmerli L et al. (2004) The PEN1 syntaxin defines a novel cellular compartment upon fungal attack and is required for the timely assembly of papillae. Mol Biol Cell 15: 5118-5129.

2. Nuhse TS, Boller T, Peck SC (2003) A plasma membrane syntaxin is phosphorylated in response to the bacterial elicitor flagellin. J Biol Chem 278: 45248-45254.

3. Sanderfoot AA, Assaad FF, Raikhel NV (2000) The Arabidopsis genome. An abundance of soluble N-ethylmaleimide-sensitive factor adaptor protein receptors. Plant Physiol 124: 1558-1569.

4. Tyrrell M, Campanoni P, Sutter JU, Pratelli R, Paneque M et al. (2007) Selective targeting of plasma membrane and tonoplast traffic by inhibitory (dominant-negative) SNARE fragments. Plant J 51: 1099-1115.

5. Lipka V, Kwon C, Panstruga R (2007) SNARE-ware: the role of SNARE-domain proteins in plant biology. Annu Rev Cell Dev Biol 23: 147-174.

6. Stein M, Dittgen J, Sanchez-Rodriguez C, Hou BH, Molina A et al. (2006) Arabidopsis PEN3/PDR8, an ATP binding cassette transporter, contributes to nonhost resistance to inappropriate pathogens that enter by direct penetration. Plant Cell 18: 731-746.

7. Lee M, Lee K, Lee J, Noh EW, Lee Y (2005) AtPDR12 contributes to lead resistance in Arabidopsis. Plant Physiol 138: 827-836.

8. Kwon C, Bednarek P, Schulze-Lefert P (2008) Secretory pathways in plant immune responses. Plant Physiol 147: 1575-1583.

9. Campbell EJ, Schenk PM, Kazan K, Penninckx IA, Anderson JP et al. (2003) Pathogen-responsive expression of a putative ATP-binding cassette transporter gene conferring resistance to the diterpenoid sclareol is regulated by multiple defense signaling pathways in Arabidopsis. Plant Physiol 133: 1272-1284.

10. Panstruga R (2005) Serpentine plant MLO proteins as entry portals for powdery mildew fungi. Biochem Soc Trans 33: 389-392.

11. Chen Z, Hartmann HA, Wu MJ, Friedman EJ, Chen JG et al. (2006) Expression analysis of the AtMLO gene family encoding plant-specific seven-transmembrane domain proteins. Plant Mol Biol 60: 583-597.

12. Consonni C, Humphry ME, Hartmann HA, Livaja M, Durner J et al. (2006) Conserved requirement for a plant host cell protein in powdery mildew pathogenesis. Nat Genet 38: 716-720.

13. Bhat RA, Miklis M, Schmelzer E, Schulze-Lefert P, Panstruga R (2005) Recruitment and interaction dynamics of plant penetration resistance components in a plasma membrane microdomain. Proc Natl Acad Sci U S A 102: 3135-3140.

14. Heese A, Hann DR, Gimenez-Ibanez S, Jones AM, He K et al. (2007) The receptor-like kinase SERK3/BAK1 is a central regulator of innate immunity in plants. Proc Natl Acad Sci U S A 104: 12217-12222.

15. Chinchilla D, Zipfel C, Robatzek S, Kemmerling B, Nurnberger T et al. (2007) A flagellin-induced complex of the receptor FLS2 and BAK1 initiates plant defence. Nature 448: 497-500.

16. Schwessinger B, Zipfel C (2008) News from the frontline: recent insights into PAMP-triggered immunity in plants. Curr Opin Plant Biol 11: 389-395.

17. Kemmerling B, Schwedt A, Rodriguez P, Mazzotta S, Frank M et al. (2007) The BRI1-associated kinase 1, BAK1, has a brassinolide-independent role in plant cell-death control. Curr Biol 17: 1116-1122.

18. Huffaker A, Ryan CA (2007) Endogenous peptide defense signals in Arabidopsis differentially amplify signaling for the innate immune response. Proc Natl Acad Sci U S A 104: 10732-10736.

19. Huffaker A, Pearce G, Ryan CA (2006) An endogenous peptide signal in Arabidopsis activates components of the innate immune response. Proc Natl Acad Sci U S A 103: 10098-10103.

20. Ryan CA, Huffaker A, Yamaguchi Y (2007) New insights into innate immunity in Arabidopsis. Cell Microbiol 9: 1902-1908.

21. Liu J, Liu X, Dai L, Wang G (2007) Recent progress in elucidating the structure, function and evolution of disease resistance genes in plants. J Genet Genomics 34: 765-776.

22. Jones JD, Dangl JL (2006) The plant immune system. Nature 444: 323-329.

23. Nawrath C, Heck S, Parinthawong N, Metraux JP (2002) EDS5, an essential component of salicylic acid-dependent signaling for disease resistance in Arabidopsis, is a member of the MATE transporter family. Plant Cell 14: 275-286.

24. Wildermuth MC, Dewdney J, Wu G, Ausubel FM (2001) Isochorismate synthase is required to synthesize salicylic acid for plant defence. Nature 414: 562-565.

25. Wang L, Tsuda K, Sato M, Cohen JD, Katagiri F et al. (2009) Arabidopsis CaM binding protein CBP60g contributes to MAMP-induced SA accumulation and is involved in disease resistance against Pseudomonas syringae. PLoS Pathog 5: e1000301.

26. Tsutsui T, Morita-Yamamuro C, Asada Y, Minami E, Shibuya N et al. (2006) Salicylic acid and a chitin elicitor both control expression of the CAD1 gene involved in the plant immunity of Arabidopsis. Biosci Biotechnol Biochem 70: 2042-2048.

27. Morita-Yamamuro C, Tsutsui T, Sato M, Yoshioka H, Tamaoki M et al. (2005) The Arabidopsis gene CAD1 controls programmed cell death in the plant immune system and encodes a protein containing a MACPF domain. Plant Cell Physiol 46: 902-912.

28. Truman W, Bennett MH, Kubigsteltig I, Turnbull C, Grant M (2007) Arabidopsis systemic immunity uses conserved defense signaling pathways and is mediated by jasmonates. Proc Natl Acad Sci U S A 104: 1075-1080.

29. Glazebrook J (2005) Contrasting mechanisms of defense against biotrophic and necrotrophic pathogens. Annual Reviews of Phytopathology 43: 205-227.

30. Glazebrook J, Chen W, Estes B, Chang HS, Nawrath C et al. (2003) Topology of the network integrating salicylate and jasmonate signal transduction derived from global expression phenotyping. Plant J 34: 217-228.

31. Schuhegger R, Nafisi M, Mansourova M, Petersen BL, Olsen CE et al. (2006) CYP71B15 (PAD3) catalyzes the final step in camalexin biosynthesis. Plant Physiol 141: 1248-1254.

32. Rauhut T, Luberacki B, Seitz HU, Glawischnig E (2009) Inducible expression of a Nep1-like protein serves as a model trigger system of camalexin biosynthesis. Phytochemistry 70: 185-189.

33. Schuhegger R, Rauhut T, Glawischnig E (2007) Regulatory variability of camalexin biosynthesis. J Plant Physiol 164: 636-644.

34. Glawischnig E, Hansen BG, Olsen CE, Halkier BA (2004) Camalexin is synthesized from indole-3-acetaldoxime, a key branching point between primary and secondary metabolism in Arabidopsis. Proc Natl Acad Sci U S A 101: 8245-8250.

35. Glawischnig E (2007) Camalexin. Phytochemistry 68: 401-406.

36. Zhao J, Last RL (1996) Coordinate regulation of the tryptophan biosynthetic pathway and indolic phytoalexin accumulation in Arabidopsis. Plant Cell 8: 2235-2244.

37. Kliebenstein DJ, Rowe HC, Denby KJ (2005) Secondary metabolites influence Arabidopsis/Botrytis interactions: variation in host production and pathogen sensitivity. Plant J 44: 25-36.

38. Shridhar S, Chattopadhyay D, Yadav G (2009) PLecDom: a program for identification and analysis of plant lectin domains. Nucleic Acids Res

39. Van Damme EJ, Barre A, Rouge P, Peumans WJ (2004) Cytoplasmic/nuclear plant lectins: a new story. Trends Plant Sci 9: 484-489.

40. Eulgem T, Rushton PJ, Robatzek S, Somssich IE (2000) The WRKY superfamily of plant transcription factors. Trends Plant Sci 5: 199-206.

41. Fujimoto SY, Ohta M, Usui A, Shinshi H, Ohme-Takagi M (2000) Arabidopsis ethylene-responsive element binding factors act as transcriptional activators or repressors of GCC box-mediated gene expression. Plant Cell 12: 393-404.

42. Libault M, Wan J, Czechowski T, Udvardi M, Stacey G (2007) Identification of 118 Arabidopsis transcription factor and 30 ubiquitin-ligase genes responding to chitin, a plant-defense elicitor. Mol Plant Microbe Interact 20: 900-911.

43. Onate-Sanchez L, Singh KB (2002) Identification of Arabidopsis ethylene-responsive element binding factors with distinct induction kinetics after pathogen infection. Plant Physiol 128: 1313-1322.

44. Chini A, Fonseca S, Fernandez G, Adie B, Chico JM et al. (2007) The JAZ family of repressors is the missing link in jasmonate signalling. Nature 448: 666-671.

45. Thines B, Katsir L, Melotto M, Niu Y, Mandaokar A et al. (2007) JAZ repressor proteins are targets of the SCF(COI1) complex during jasmonate signalling. Nature 448: 661-665.

46. Lorenzo O, Chico JM, Sanchez-Serrano JJ, Solano R (2004) JASMONATE-INSENSITIVE1 encodes a MYC transcription factor essential to discriminate between different jasmonate-regulated defense responses in Arabidopsis. Plant Cell 16: 1938-1950.

47. Zheng MS, Takahashi H, Miyazaki A, Hamamoto H, Shah J et al. (2004) Up-regulation of Arabidopsis thaliana NHL10 in the hypersensitive response to Cucumber mosaic virus infection and in senescing leaves is controlled by signalling pathways that differ in salicylate involvement. Planta 218: 740-750.

48. Gopalan S, Wei W, He SY (1996) hrp gene-dependent induction of hin1: a plant gene activated rapidly by both harpins and the avrPto gene-mediated signal. Plant J 10: 591-600.

49. Slocum RD, Kaur-Sawhney R, Galston AW (1984) The physiology and biochemistry of polyamines in plants. Arch Biochem Biophys 235: 283-303.

50. Lecourieux D, Mazars C, Pauly N, Ranjeva R, Pugin A (2002) Analysis and effects of cytosolic free calcium increases in response to elicitors in Nicotiana plumbaginifolia cells. Plant Cell 14: 2627-2641.

51. Grant M, Brown I, Adams S, Knight M, Ainslie A et al. (2000) The RPM1 plant disease resistance gene facilitates a rapid and sustained increase in cytosolic calcium that is necessary for the oxidative burst and hypersensitive cell death. Plant J 23: 441-450.

52. Gust AA, Biswas R, Lenz HD, Rauhut T, Ranf S et al. (2007) Bacteria-derived peptidoglycans constitute pathogen-associated molecular patterns triggering innate immunity in Arabidopsis. J Biol Chem 282: 32338-32348.
