# Supplementary material for: The Arabidopsis Wall Associated Kinase-Like 10 Gene Encodes a Functional Guanylyl Cyclase and Is Co-Expressed with Pathogen Defense Related Genes
Source: PLoS One. 2010 Jan 26;5(1):e8904. doi: 10.1371/journal.pone.0008904 (PMC2811198; doi:10.1371/journal.pone.0008904)
Supplement: Text S2 — Description of microarray experiments. (0.03 MB DOC) [file pone.0008904.s005.doc]

**Text S2. Description of microarray experiments**

**Chitooctaose treatment of wiltype and chitin receptor mutants (GSE8319).**

Two-week-old hydroponically grown seedlings were treated with chitooctaose at a final concentration of 1 µm for 30 minutes.

Genotypes:Wild type Col-0 and *chitin-elicitor receptor kinase* (*cerk1*, AT3G21630) mutant.

(n=3)

**Bacterial elongation factor Tu (EF-Tu)** (**E-MEXP-547)**

Fourteen day old hydroponically grown Arabidopsis seedlings were treated (by addition to growth medium) with the bacterial elf26 MAMP derived elicitor peptide at a final concentrationof 10 µM for 0 (control) 30 and 60 minutes.

Genotypes: Landsberg erecta

(n=2)

**Oligogalacturonide treatment of Arabidopsis seedlings (NASC-409)**

Ten day old Arabidopsis seedlings were treated with 50 ug/ml oligogalacturonides (OGs), (cell wall fragments released by the activity of fungal polygalacturonases), 1 µM flg22, or an equivalent volume of water (control). Tissue was harvested and transcript levels were determined 1 and 3 hours after treatment (hat).

Genotype: Col-0

(n=3)

**Response to bacterial-(HrpZ) and oomycete-(NPP1) derived elicitors (NASC-122 / GSE5615)**

The leaves of five week old plants were infiltrated with either the bacterial-HrpZ (1 µM) or oomycete-(NPP1) (2 µM) derived elicitors and leaf tissue was harvested at 1 and 4 hat.

Genotype: Col-0

(n=3)

**Syringolin A (E-MEXP-739)**

The surface of rosette leaves of 12-14 day old soil grown plants were sprayed with a 20 uM syringolin A (sly A) solution or a control buffer solution and primary leaf tissue was collected and pooled for RNA extraction at 8 and 12 hat.

Genotype: Col-0

(n=3)

***Phytophthora infestans* (NASC-123)**

Spores of *P.infestans* (5x10^5^) were applied to leaf surfaces of 5-week old Arabidopsis leaves and leaf tissue was extracted at 6, 12 and 24 hat.

Genotype: Col-0

(n=3)

***Blumeria graminis*** *f.sp. hordei* **infection (GSE12856)**

Leaves of six week old Arabidopsis plants were spray inoculated with the non-host biotrophic fungus *B. graminis* f.sp. hordei (*B. graminis* *h*): for 12 h. RNA was extracted from rosette leaves 12 hat.

Genotype: Col-0

(n=3)

***Erysiphe cichoracearum* (GSE431)**

The powdery mildew, E. cichoracearum, was cultured on squash for 10-12 days and was then applied to the leaves of three week old Arabidopsis plants using 1.3 m settling towers. RNA was extracted 3 days after treatment (dat).

Genotype: Col-0

(n=4)

***Golovinomyces orontii* infection time course (GSE13739)**

Entire leaves of four week old Arabidopsis plants were spray inoculated with *G. orontii* and whole leaf tissue was harvested at time 0, 6hat, 1, 3, 5 and 7 dat for RNA extraction

Genotypes: Col-0 and enhanced disease susceptibility (*eds)-16*, a null isochorismate synthase 1 (*ics1*) mutant (At1g74710).

(n=4)

***Botrytis cinerea* infection (NASC-167)**

Fully expanded rosette leaves of four week old Arabidopsis plants were inoculated with four 5µl drops of a *B. cinerea* spore solution (5x10^5^/ml). Leaf tissue was harvested 18 and 48 hat for RNA extraction.

Genotype: Col-0

(n=3)

***Pseudomonas syringae* time course (NASC-120)**

Five-week old Arabidopsis plants were infiltrated with 10^8^ cfu/ml of various *P. syringae* strains including the virulent pv. tomato DC3000 (*Pst*), the avirulent pv. tomato *avrRpm1* (*Pst avrRpm1*) and the non-host pv. *Phaseolicola* (*Psph*). Leaf tissue was harvested at 2, 6 and 24 hat.

Genotype: Col-0

(n=3)

**Benzothiadiazole S-methylester (BTH) treatment (NASC-392)**

Four week old Arabidopsis plant leaf surfaces were sprayed with 60uM of the functional synthetic SA analogue BTH and leaf tissue was collected at 0, 8, and 24 hat. The fold change in genes expression was determined for BTH treatment Vs untreated controls (Col-0) and BTH treatment of *npr1* mutants Vs BTH treatment of Col-0.

Genotypes: Col-0 and *npr1-1* mutant.

(n = 3)

**Methyl jasmonate (meJA) treatment (NASC 415)**

Six week old plants were sprayed with water containing 250 µM meJA and 0.1% (v/v) ethanol, 5.5 hrs into the light cycle and 25 hat RNA was extracted from aerial tissue for comparison of gene expression profiles

Genotype: Col-0

(n=3)

**Cyclohexamide (CHX) treatment (NASC-189)**

Seven day old seedlings were treated with 10uM CHX for three hours.

Genotype: Col-0

(n=2)
